# Guidelines for Seizure Prophylaxis in Patients Undergoing Supratentorial Neurosurgery: A Statement for Healthcare Professionals from the Neurocritical Care Society

**DOI:** 10.1007/s12028-026-02522-2

**Published:** 2026-05-05

**Authors:** A. Shaun Rowe, Jamie Ullman, Emily L. Johnson, Emily J. Gilmore, DaiWai Olson, Appaji Rayi, Eljim Tesoro, Yuhong Yuan, Sahar Zafar, Jennifer A. Frontera

**Affiliations:** 1 Department of Clinical Pharmacy and Translational Science, University of Tennessee Health Science Center College of Pharmacy, Knoxville, Tennessee USA; 2 Department of Neurosurgery, Zucker School of Medicine at Hofstra/Northwell, Hempstead, NewYork USA; 3 Department of Neurology, Johns Hopkins School of Medicine, Baltimore, Maryland USA; 4 Department of Neurology, Yale School of Medicine, New Haven, Connecticut USA; 5 Department of Neurology, UT Southwestern, Dallas, Texas USA; 6 Department of Neurology, Charleston Area Medical Center, Charleston, West Virginia USA; 7 Department of Pharmacy Practice, Retzky College of Pharmacy, University of Illinois Chicago, Chicago, Illinois USA; 8 Department of Medicine, London Health Science Centre, London, Ontario Canada; 9 Department of Neurology, Harvard Medical School, Boston, Massachusetts USA; 10 Department of Neurology, New York University Grossman School of Medicine, New York, USA

**Keywords:** Supratentorial surgery, Craniotomy, Prophylaxis, Seizure, Anti-seizure medication, Levetiracetam, Phenytoin

## Abstract

**Background:**

There is significant heterogeneity related to the use of prophylactic antiseizure medications (ASM) following supratentorial craniotomy.

**Methods:**

We conducted a systematic review and meta-analysis assessing ASM primary prophylaxis in adults hospitalized following supratentorial neurosurgery with no prior seizure history. The following population, intervention, comparator, and outcome (PICO) questions were assessed: (1) Should ASM versus no ASM be used as seizure prophylaxis in adult patients undergoing supratentorial neurosurgery? (2) If an ASM is used, should levetiracetam (LEV) or phenytoin/fosphenytoin (PHT) be preferentially used? and (3) Should a long (> 7 days) versus short (≤ 7 days) duration of prophylaxis be used? The main outcomes were early seizure (≤ 14 days), late seizures (> 14 days), adverse events, mortality, and functional and cognitive outcomes. We utilized Grading of Recommendations Assessment, Development and Evaluation (GRADE) methodology to generate recommendations.

**Results:**

The initial literature search yielded 1988 articles, and 16 formed the basis of the recommendations. PICO 1: while meta-analysis of randomized controlled trials (RCTs) demonstrated a significant benefit for early seizure prevention, meta-analyses including all study designs was nonsignificant. Further, there were no differences in late seizure or mortality rates, and there was a trend toward higher adverse event rates with ASM. PICO 2: LEV was associated with significantly lower early seizure rates than PHT, and there were trends toward fewer late seizures and adverse events with LEV. PICO 3: only three studies examined the duration of ASM treatment, and there was no significant difference in seizure events between subjects treated for a short versus long duration.

**Conclusions:**

We suggest that either prophylactic ASM or no ASM be used for seizure prophylaxis in patients undergoing supratentorial neurosurgery (*conditional recommendation, low quality of evidence*). If an ASM is used, we suggest LEV over PHT (*conditional recommendation, very low quality of evidence*) for a short duration (*conditional recommendation, very low quality of evidence*)*.*

**Supplementary Information:**

The online version contains supplementary material available at 10.1007/s12028-026-02522-2.

## Introduction

Patients undergoing supratentorial neurosurgery are often treated with an anti-seizure medication (ASM) as primary prevention against seizures. The risk of early postoperative seizures has been reported to be 15–20% [1, 2]. However, this risk varies by type of surgery, underlying pathology, and previous history of seizures [2, 3]. In addition, there is large variation in clinical practice as to the specific ASM agent, duration of treatment, and dose of treatment. Because of the relatively high risk of postoperative seizures and the variance in practice, this guideline sought to establish guidance for healthcare professionals treating such patients. The primary questions we aimed to address were: (1) Should prophylactic ASM versus no ASM be used in patients hospitalized for supratentorial neurosurgery? (2) If an ASM is used, should levetiracetam (LEV) or phenytoin /fosphenytoin (PHT) be preferentially prescribed? and (3) If an ASM is used, should a short (≤ 7 days) versus longer (> 7 days) duration of therapy be used?

### ***Methods***

This guideline was developed in accordance with Grading of Recommendations Assessment, Development and Evaluation (GRADE) methodology [4, 5] and both panel co-chairs (JAF and ASR) completed GRADE workshop training [6]. The GRADE methodology requires the guideline panel to make independent decisions concerning the certainty of evidence and the strength of the recommendation. Thus, even when there is low or very-low quality of evidence, the panel can make a recommendation on the question. Throughout this guideline, the panel has made recommendations on the basis of the evidence that is available at the time of writing. We further describe how we came to our level of certainty and strength of recommendations in the ‘Risk of Bias’ and ‘Certainty of Evidence Evaluation and Development of Recommendations’ sections, respectively.

### Panel Composition

The Seizure Prophylaxis Guideline panel was formed in October 2019 and consists of nine members, including six physicians, two pharmacists, and one nurse with subspecialty experience in neurocritical care, seizure management, trauma, and neurosurgery. In addition, a GRADE statistician (YY) performed statistical analyses. The panel consisted of six women and four men of diverse racial/ethnic backgrounds (Asian, South Asian, white, and Hispanic).

### Disclosure and Management of Potential Conflicts of Interest

All panel members were required to comply with standard conflict of interest and commercial relationship disclosures, including review of any financial, intellectual, or other relationships that may be construed as a possible conflict of interest. The chairs of the Neurocritical Care Society Guideline Committee that oversees the Seizure Prophylaxis Guideline Panel were responsible for vetting any potential conflicts of interest. All members of the Seizure Prophylaxis Guideline Panel were determined to be free of conflicts of interest.

### PICO Generation

Three specific questions were addressed for this guideline following the population, intervention, comparison, and outcomes (PICO) format [7]. These questions were developed to address the overall goal set forth by the Neurocritical Care Society (NCS) Guidelines Committee. Final questions were agreed on by all guideline panel members. The PICOs are as follows: (1) Should ASM versus no ASM be used in patients hospitalized for supratentorial neurosurgery with no history of clinical or electrographic seizures? (2) If an ASM is used, should LEV or PHT be preferentially used for supratentorial neurosurgery patients with no history of clinical or electrographic seizures? and (3) If an ASM is used, should a long (> 7 days) versus short (≤ 7 days) duration of prophylaxis be used for supratentorial neurosurgery patients with no history of clinical or electrographic seizures?

The outcomes categorized as “critical” (indicating the highest level of importance) included early seizure (either clinical or electrographic) occurring within 14 days of supratentorial neurosurgery, late seizure (either clinical or electrographic) occurring > 14 days from supratentorial neurosurgery, and adverse events associated with ASM use. A 14-day cut-off was used to distinguish early from late seizures because literature is inconsistent in the definition, with some studies defining early seizures as those within 7 days and others defining early seizures within 14 days. Since neither the 7- nor the 14-day timeframe is based on a clear biological rationale, we selected the 14-day window to capture the widest range of available evidence. Additional outcomes including mortality, functional (e.g., modified Rankin Scale scores [8], Glasgow Outcome Scale [9]) and cognitive outcomes were rated as “important.” In this guideline, LEV was used as a comparator and as a representative of the newer generation of Antiseizure Drug (ASD) medications and phenytoin/fosphenytoin were chosen as representatives of older generation ASD medications. The goal was to provide as homogenous of a comparison as possible. We recognize this as a potential limitation and encourage new studies valuating the use of other, newer generation ASDs.2

### Study Population

This guideline pertains to hospitalized patients undergoing supratentorial neurosurgery for any reason or underlying pathology, including but not limited to tumors, vascular lesions, and traumatic lesions. The target population is limited to individuals without a prior history of seizure (clinical or electrographic) or ASM use prior to index supratentorial neurosurgery. The authors chose to exclude infratentorial (posterior fossa) surgery owing to a low risk of seizure. This choice is based upon standard knowledge. While there has been some attention to the role of the cerebellum in epilepsy, there are no studies available from our search discussing seizure incidence after posterior fossa surgery [10, 11]. Additional guidelines for ASM prophylaxis created for hospitalized patients with nontraumatic subarachnoid hemorrhage, intracerebral hemorrhage [12], and traumatic brain injury [13] are published separately.

### Inclusion and Exclusion Criteria

Studies could be included if the following criteria were met: the paper addressed prophylactic ASM use, included an adult population (aged ≥ 18 years) hospitalized with supratentorial neurosurgery, and if data were available on the primary outcomes of interest (early seizure, late seizure, adverse events, mortality, functional outcomes, or cognitive outcomes). Articles were excluded if they involved patients with a history of seizure, epilepsy, or chronic ASM use prior to supratentorial neurosurgery; were not published in English; were non-human studies; were case series with < 10 patients; evaluated a pediatric population; or did not assess an outcome of interest. We excluded gray literature including abstracts, conference proceedings, and non-peer reviewed articles, as well as review articles and meta-analyses.

### Search Strategy

A search of articles was conducted by an independent medical librarian from 1 January 1946 to 7 October 2020 using PubMed, Medline, Embase, Emcare, and Cochrane databases (Supplementary Table 1). Additional literature searches were performed by panel members between 7 October 2020 and 9 October 2024 to capture more recently published articles. Search terms included: “seizure,” “antiepileptic medication,” “antiseizure medication,” “levetiracetam,” “Keppra,” “lacosamide,” “Vimpat,” “phenytoin,” “Dilantin,” “fosphenytoin,” “Cerebyx,” “valproic acid,” “Depakote,” “carbamazepine,” “lamotrigine,” “prophylaxis,” “prevention,” “prophylactic,” “craniectomy,” “craniotomy,” “trephining,” “neurosurgery,” “neurosurgical,” “supratentorial,” mortality,” “death,” “functional outcome,” “function,” “modified Rankin,” “Glasgow Outcome score,” “cognition,” “cognitive,” “disability,” “activities of daily living,” “outcome,” “adverse events,” and “side effects.” Reference lists of published articles, review articles, and meta-analyses were also screened to identify additional articles.

### Study Screening and Data Collection

Two reviewers independently screened each article title and abstract to determine inclusion eligibility. Reviewers were blinded to each other’s verdicts during screening. Full-text screening by two independent reviewers was performed on articles that passed the initial level of review. Screening was performed using DistillerSR software (Ottawa, Ontario, Canada) and all conflicts were adjudicated between reviewers prior to study inclusion. Data were extracted into a standardized tool and classified as randomized controlled trials versus nonrandomized studies, which could be observational studies using retrospective cohort, prospective cohort, cross-sectional, or case series designs.

### Risk of Bias and Certainty of Evidence Evaluation

Risk of bias was assessed using the Cochrane Risk of Bias 2 (RoB-2) tool [14] for randomized trials and the Risk of Bias Instrument for Non-randomized Studies-of Interventions (ROBINS-I) tool [15] for nonrandomized studies. These tools were selected on the basis of recommendations from GRADE and the types of articles evaluated. Final risk-of-bias scores were adjudicated by two reviewers who were blinded to each other’s scores prior to adjudication. RoB-2 scoring specifically addresses bias concerns related to randomization, deviation from intended interventions, missing outcome data, measurement of outcome, and selection of the reported result. The ROBINS-I assessment accounted for bias in confounding, patient selection, classification of interventions, deviations from intended interventions, missing data, measurement of outcomes, and selection of the reported result.

**Fig. 1:**
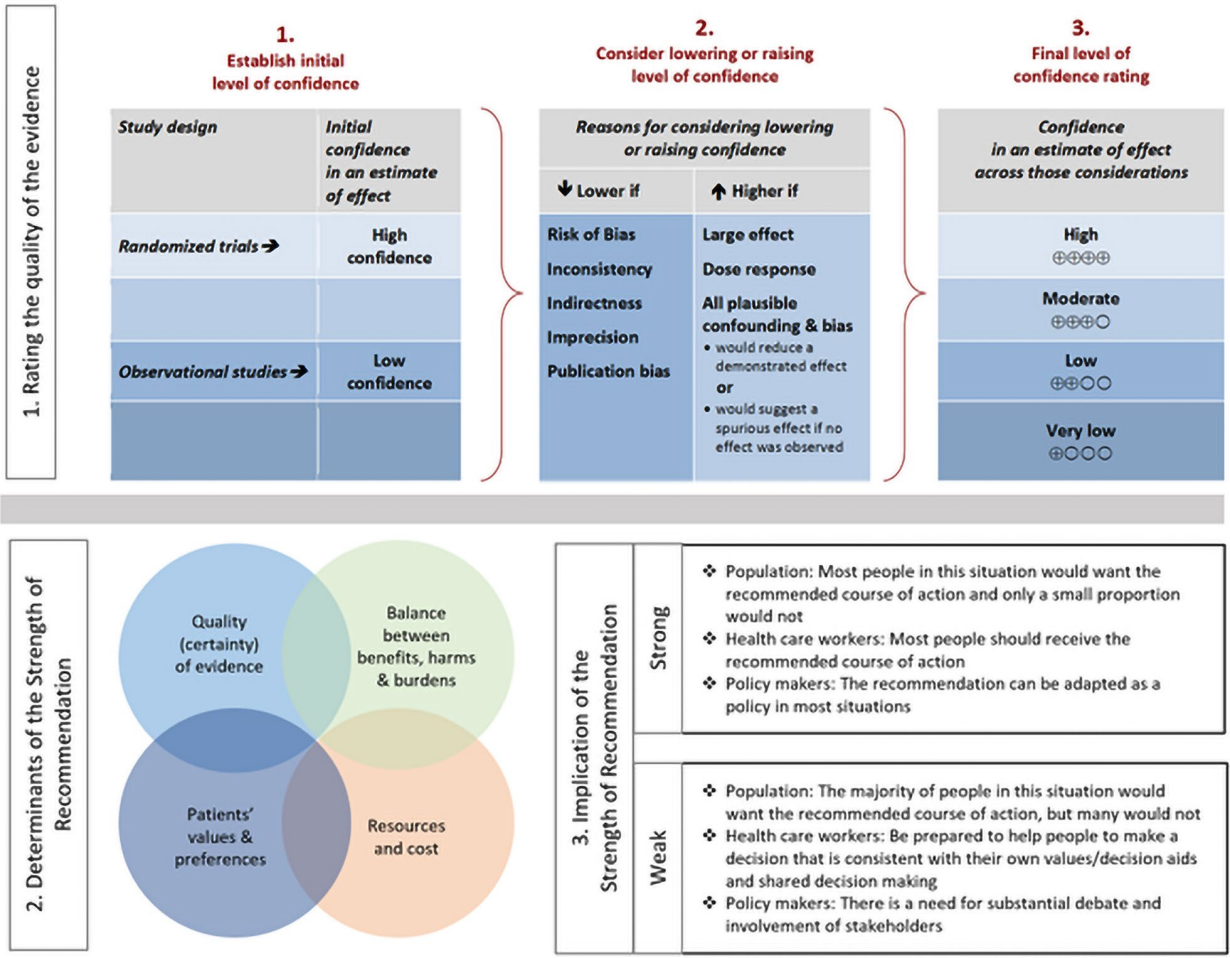
GRADE methodology for rating certainty of evidence, level of confidence, and determining the strength of recommendation. Strong recommendations use the term “recommend,” while conditional recommendations use the term “suggest.” Unrestricted use of this figure was granted by the US GRADE Network. GRADE, Grading of Recommendations Assessment, Development and Evaluation

The certainty of evidence assessment was performed using GRADEPro GDT software (McMaster University and Evidence Prime Inc.) according to GRADE methodology [16]. In brief, studies that address a specific outcome of interest can be assessed as a group to determine the certainty with which the evidence leads the panel to make a recommendation. The certainty of evidence may be reduced by the risk of bias, inconsistency (heterogeneity across different studies, typically signified by high *I*^2^ values), indirectness (how closely the studies pertain to the PICO), imprecision (unclear effect size owing to low event rates, small sample sizes, or wide confidence intervals), and publication bias. The certainty of evidence could be increased by a large effect size, a dose–response gradient, or residual confounding that favors the comparator. A final level of confidence rating is generated from this process ranging from very-low to high confidence in the estimate of effect. All guideline panel members were involved with determination of the certainty of evidence and final recommendations were agreed upon by all panel members (Fig. 1).

### Statistical Analyses

All analyses were outcome based and performed by one study statistician (YY). For each outcome of interest (early seizure, late seizure, adverse events, functional and cognitive outcomes, and mortality), we stratified the analysis by ASM type and also by study design (randomized versus nonrandomized studies) and tested their differences. The summary statistic used for dichotomous data was relative risk, and the mean difference or standardized mean difference was used, when applicable, for continuous data. Studies that reported adjusted odds ratios (ORs) were pooled using the method of inverse variance. All meta-analyses were conducted using random-effects models. Substantial heterogeneity was defined as *I*^2^ ≥ 50%. All analyses are presented in forest plots and were performed using Revman 5.4 software (Cochrane, London, UK).

### Development of Recommendations

Assessments of judgement for each PICO were performed utilizing GRADEPro GDP software (McMaster University and Evidence Prime Inc.). Final recommendations were based on consideration of the importance of the PICO, the certainty and confidence level of the evidence, the balance between the desirable and undesirable effects of the intervention, patient values, and the acceptability and feasibility of the recommendation following the GRADE Evidence-Decision framework (Fig. 1). Consensus of all panel members was required for final recommendations. Independent members of the guideline committees from the Neurocritical Care Society reviewed all recommendations. Strong recommendations, which imply that the majority of stakeholders would want to adopt the prescribed guidance and policy-makers may utilize the guideline in most situations, are indicated by the phrase “we recommend.” Conditional recommendations, which imply that most stakeholders would want to adopt the recommendation, though many might not, and that shared decision making between patient and practitioner is likely required, are indicated by the verbiage “we suggest.” The overall certainty (quality) of evidence was averaged across outcomes for each PICO and could be categorized as very low, low, moderate, or high. The limitations in the current body of literature and proposals for future avenues of research are discussed with each PICO.

The “In Our Practice” section follows the formal GRADE-based recommendations and justifications. This section highlights current practices that might not be specifically covered in the recommendations. The pragmatic details of this section were arrived at after panel discussion and represent expert consensus. A caveat to this section is that panel members primarily represent academic centers and reflect current practice in the USA. As such, these suggestions may not be generalizable to all settings.

## Results

A summary of recommendations is presented in Table 1. The initial literature search yielded 1988 articles, of which 16 formed the basis of the recommendations, and 15 were included in meta-analyses. A summary of sample size, design, surgical indication, and ASM dosing of the studies included in the metanalyses can be found in Table 2. PRISMA recommendations were followed while conducting the systematic review (Supplementary Fig. 1).

**Table 1:** Summary of recommendations for seizure prophylaxis for supratentorial neurosurgery

jlvt	Recommendation	Level of recommendation, quality (certainty) of evidence	Justification
PICO 1	Should *anti-seizure medication versus no anti-seizure medication* be used in patients hospitalized for supratentorial neurosurgery in patients with no history of clinical or electrographic seizures?		
Recommendation 1	We suggest that clinicians may choose to use either ASM or not use ASM for seizure prevention in patients undergoing supratentorial neurosurgery, as the balance of benefits and harms is uncertain	Conditional recommendation, low certainty of evidence	As it relates to early seizure, late seizure, and mortality, there was no positive or negative effect detected with ASM used as seizure prophylaxis. There was a trend toward more adverse events in patients treated with ASM, but it did not reach statistical significance
PICO 2	If an anti-seizure medication is used, should *LEV or PHT* be preferentially used for supratentorial neurosurgery in patients with no history of clinical or electrographic seizures?		
Recommendation 2	When prophylactic ASM is used for supratentorial craniotomy, we suggest LEV over PHT	Conditional recommendation, very-low certainty of evidence	As it relates to early seizure, LEV was better than PHT. There was no difference in LEV and PHT for late seizure and adverse events
PICO 3	If an anti-seizure medication is used, should a *long (*> *7 days) versus short (*≤ *7 days) duration* of prophylaxis be used for supratentorial neurosurgery in patients with no history of clinical or electrographic seizures?		
Recommendation 3	If a prophylactic ASM is used for supratentorial craniotomy, we suggest a short duration (≤ 7 days) vs. a longer duration (> 7 days) of use	Conditional recommendation, very-low certainty of evidence	There was very limited evidence related to the effect of duration of ASM on early seizure, late seizure, and adverse events. Our recommendation assumed the potential for increased adverse events and the lack of difference in positive outcomes

Per GRADE methodology, “strong” recommendations use the term “recommend” and “conditional” recommendations use the term “suggest” NCS, neurocritical care society; ASM, anti-seizure medication

**Table 2:** Summary of size, surgical indication, and ASM dosage for studies included in meta-analyses

Citation	Title	Groups	Methodology	Surgical indication	Intervention
Al-Dorzi [17]	Incidence, risk factors, and outcomes of seizures occurring after craniotomy for primary brain tumor resection	ASM (*N* = 32) No ASM (*N* = 92)	Nonrandomized controlled trial	Intracranial tumor (low-grade glioma, high-grade glioma, meningioma, others)	PHT: no dosage provided Carbamazepine: no dosage provided Other: no dosage provided
Battaglia [21]	Is there any benefit from short-term perioperative antiepileptic prophylaxis in patients with chronic subdural haematoma?	ASM (*N* = 48) No ASM (*N* = 51)	Nonrandomized controlled trial	Chronic subdural hematoma	LEV: 500 mg twice daily
Foy [26]	Do prophylactic anticonvulsant drugs alter the pattern of seizures after craniotomy?	ASM (*N* = 217) No ASM (*N* = 59)	Randomized controlled trial	Aneurysm, arteriovenous malformation. Spontaneous hematoma, abscess, meningioma, and benign tumors	Carbamazepine: loaded with 200 mg every 6h for 24h prior to surgery followed by 200 mg every 8 h PHT: 15 mg/kg 24h prior to surgery followed by 100 mg every 8h
Fuller [37]	Tolerability, safety, and side effects of levetiracetam versus phenytoin in intravenous and total prophylactic regimen among craniotomy patients: a prospective randomized study	LEV (*N* = 36) PHT (*N* = 38)	Randomized controlled trial	Primary brain tumor, secondary brain metastasis, extraaxial malignancy local intracranial invasion, meningioma, cerebral abscess, subdural hematoma/epidural hematoma, intracerebral hemorrhage/subarachnoid hemorrhage, aneurysm clip	LEV: 250 mg to 500 mg twice daily PHT: 300 mg daily
Garbossa [20]	A retrospective two-center study of antiepileptic prophylaxis in patients with surgically treated high-grade gliomas	LEV (*N* = 43) No ASM (*N* = 48)	Nonrandomized controlled trial	High-grade glioma	LEV: 500 mg twice daily with stated goal of achievement of LEV serum levels above 15 μg/mL prior to surgery. No information about titration regimen
Hohne [40]	The risk of hypotension and seizures in patients receiving prophylactic anti-epileptic drugs for sup	LEV (*N* = 40) PHT (*N* = 41)	Nonrandomized controlled trial	Glioblastoma multiforme, meningioma, metastasis, astrocytoma, low-grade glioma	LEV: no dosage provided PHT: no dosage provided
Iuchi [38]	Levetiracetam versus phenytoin for seizure prophylaxis during and early after craniotomy for brain tumours: a phase II prospective, randomized study	LEV (*N* = 73) PHT (*N* = 73)	Randomized controlled trial	Glioma (grade I, II, III, IV), metastasis, meningioma, others	LEV: 500 mg twice daily until postoperative day 7 PHT: 15–18 mg/kg followed by 125 mg every 12h until postoperative day 7
Lavergne [19]	Efficacy of antiseizure prophylaxis in chronic subdural hematoma: a cohort study on routinely collected health data	ASM (*N* = 30) No ASM (*N* = 90)	Nonrandomized controlled trial	Chronic subdural hematoma	PHT: no dosage provided LEV: no dosage provided
Lee [22]	Prophylactic anticonvulsants for prevention of immediate and early postcraniotomy seizures	ASM (*N* = 189) No ASM (*N* = 185)	Randomized controlled trial	Meningioma, aneurysm, glioma, hypertensive hematoma, arteriovenous malformation, neoplasm, head injury	PHT: 15 mg/kg bolus prior to surgery followed by 5–6 mg/kg/day in three divided doses
Liang [25]	Prophylactic levetiracetam for seizure control after cranioplasty: a multicenter prospective controlled study	ASM (*N* = 97) No ASM (*N* = 100)	Randomized controlled trial	Closed head trauma or stroke	LEV: 500 mg/day for patients weighing < 60 kg or 750 mg/day for patients weighing 60 kg for 48h before the operation and 1000 mg/day for patients weighing < 60 kg or patients weighing 1500 mg/day for 60 kg from 8–12h to 24 weeks after the operation No ASM: no medication unless seizure
Milligan [39]	Efficacy and tolerability of levetiracetam versus phenytoin after supratentorial neurosurgery	LEV (*N* = 105) PHT (*N* = 210)	Nonrandomized controlled trial	Infection, primary brain tumor, metastatic tumor, meningioma, hemorrhage, vascular malformation, trauma, other	LEV: 500 mg to 3000 mg per day PHT: 300 to 800 mg per day
North [24]	Phenytoin and postoperative epilepsy: a double-blind study	ASM (*N* = 140) No ASM (*N* = 141)	Randomized controlled trial	No information on indication for craniotomy	PHT: 250 mg twice daily in the recovery room followed by 100 mg three times daily
Pradhanang [27]	Prophylactic use of antiepileptic drug (phenytoin) in preventing early postoperative seizures in patients with chronic subdural hematoma: a randomized control trial	ASM (*N* = 25) No ASM (*N* = 27)	Randomized controlled trial	Chronic subdural hematoma	PHT: load 17 mg/kg, followed by 100 mg three times daily
Wu [23]	A prospective randomized trial of peri-operative seizure prophylaxis in patients with intraparenchymal brain tumors	ASM (*N* = 62) No ASM (*N* = 61)	Randomized controlled trial	Intraparenchymal, supratentorial brain tumors	PHT: load 15 mg/kg in the operating room prior to commencing the craniotomy, followed by 100 mg every 8h
Yeap [18]	Postcranioplasty seizures following decompressive craniectomy and seizure prophylaxis: a retrospective analysis at a single institution	ASM (*N* = 56) No ASM (*N* = 280)	Nonrandomized controlled trial	Decompressive craniectomy for intracerebral hemorrhage or vascular lesions, infarction, infection, trauma, or tumor	PHT: 15–20 mg/g bolus prior to closure of cranioplasty, followed by 100 mg 3–4 times a day for 3 days, and then changed to 300 mg orally once a day for the following 4 days Sodium valproate: 10–15 mg/kg bolus before wound closure, then 10–60 mg/kg daily for 3 days, and then orally for the following 4 days LEV: 500 mg bolus before wound closure, then 500 mg twice daily

*PICO 1: Should anti-seizure medication versus no anti-seizure medication be used in adult patients hospitalized for supratentorial neurosurgery with no history of clinical or electrographic seizures?*

**Fig. 2:**
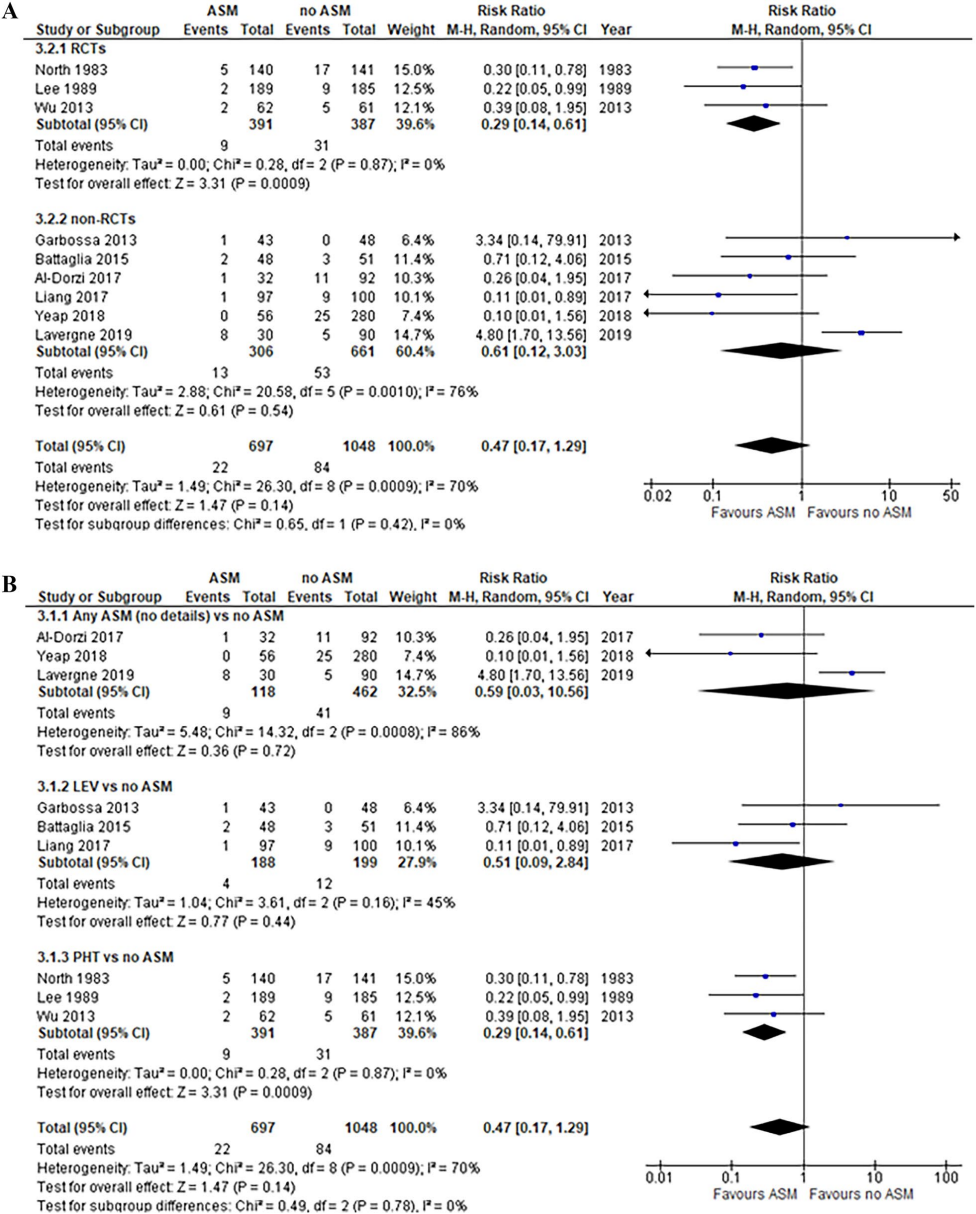
Meta-analysis (using a random-effects model) of early-seizure outcomes among patients undergoing supratentorial neurosurgery stratified by **A** randomized versus nonrandomized study design and **B** ASM type

**Fig. 3:**
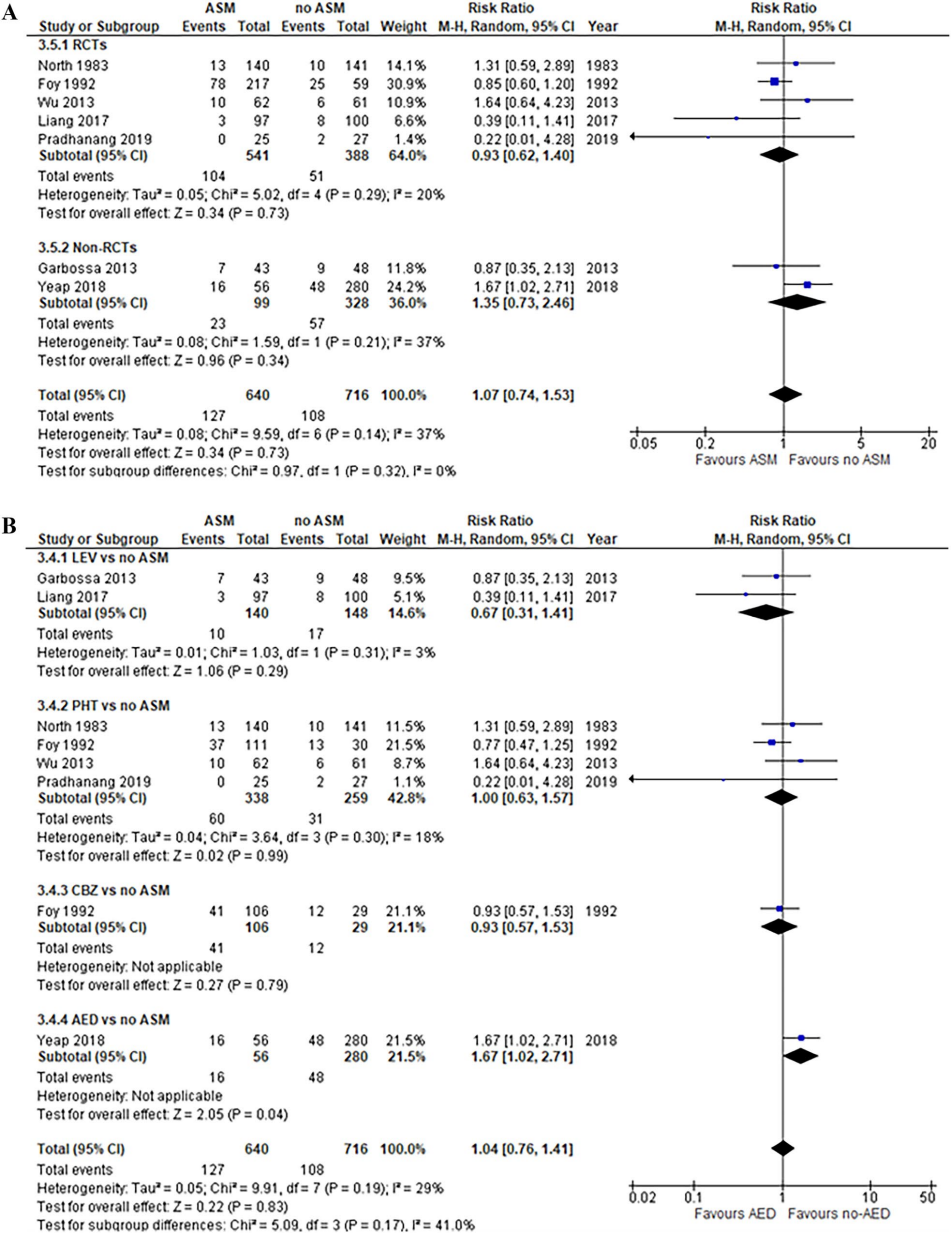
Meta-analysis (using a random-effects model) of late-seizure outcomes among patients undergoing supratentorial neurosurgery stratified by **A** randomized versus nonrandomized study design and **B** ASM type. When stratified by ASM type, the control group for one study [26] that had three arms (PHT, carbamazepine, and no ASM) was split in half to avoid double counting

- To prevent early seizures (≤ 14 days from neurosurgery or during hospitalization):

Nine studies including 1745 patients compared short-term seizure occurrence in patients treated with ASMs versus no ASMs [17–25]. Of these nine studies, three were randomized controlled trials of PHT versus no ASM and included 778 patients [22–24]. Three nonrandomized studies compared any ASM versus no ASM (*N* = 580) [17–19], while another three nonrandomized trials compared LEV versus no ASM (*N* = 190) [20, 21, 25]. The pooled analysis of all nine studies found no differences in short-term seizure prevention between patients treated with ASM versus no ASM (risk ratio [RR] 0.47, 95% confidence interval [CI] 0.17–1.29, *p* = 0.14) (Fig. 2A and B). There was significant heterogeneity across studies (*I*^2^ = 70%, *p* = 0.0009), but no significant heterogeneity between subgroups of RCT vs. non-RCT (*I*^2^ = 0%, *p* = 0.42) or between different ASM types (*I*^2^ = 0%, *p* = 0.78). However, in a meta-analysis including only RCTs, there was a significant reduction in early seizures with ASM use (all studies used PHT; RR 0.29, 95% CI 0.14–0.61, *p* = 0.0009, *I*^2^ = 0%) (Fig. 2A). By contrast, a meta-analysis of only non-RCTs showed no benefit of ASM for early seizure prevention, though there was significant heterogeneity amongst different non-RCT studies (*I*^2^ = 76%, *p* = 0.001). When stratifying the analysis by ASM type, there was a significant reduction in early seizure risk with PHT, but this was not seen with LEV or unspecified ASM. This finding reflects the fact that the RCTs utilized only PHT, while the more heterogeneous non-RCTs used LEV and other ASMs (Fig. 2B).

b. To prevent late seizures (> 14 days from neurosurgery, or post-hospitalization):

Seven studies including 1356 patients compared late-seizure outcomes. One of the studies [26] included three arms, comparing PHT, carbamazepine, and no ASM. Patients were treated and monitored for up to 24 months for the occurrence of clinical seizures. For this study with three arms, the control arm was split for the individual ASM versus no ASM analyses to avoid double-counting patients. Four analyses (*N* = 597) compared patients treated with PHT versus no ASM [23, 24, 26, 27]: one (*N* = 132) compared carbamazepine versus no ASM [26], two (*N* = 288) compared LEV versus no ASM [20, 25], and one (*N* = 336) compared any ASM versus no ASM [18]. The late clinical seizure follow-up time in these studies was between 6 and 24 months. The pooled analysis of the 1356 patients in the seven studies found no difference in long-term seizure outcome (RR 1.07, 95% CI 0.74–1.53, *I*^2^ = 37%, *p* = 0.73) (Fig. 3A and B). There was no significant heterogeneity across trials (*I*^2^ = 37%, *p* = 0.14), nor among subgroups (RCT vs. non-RCT or ASM type). Differences between the PHT, LEV, and carbamazepine versus control groups were not significant in pooled analyses. Only the subgroup containing a single study of any ASM versus no ASM (*N* = 336) found a difference between the groups, favoring no treatment with ASM (RR of seizure in ASM-treated group 1.67, 95% CI 1.02–2.71, *p* = 0.04). One retrospective study of 282 patients (not included in meta-analyses owing to lack of control group) found clinical seizure occurrence in 17% of patients who had undergone supratentorial neurosurgery and received immediate ASM prophylaxis [28]. Clinical seizures occurred at a median of 120 days (range 1–710 days) after neurosurgery (at which time many patients had discontinued ASM), indicating the risk of postoperative seizure remains elevated for many months after neurosurgery.

**Fig. 4:**
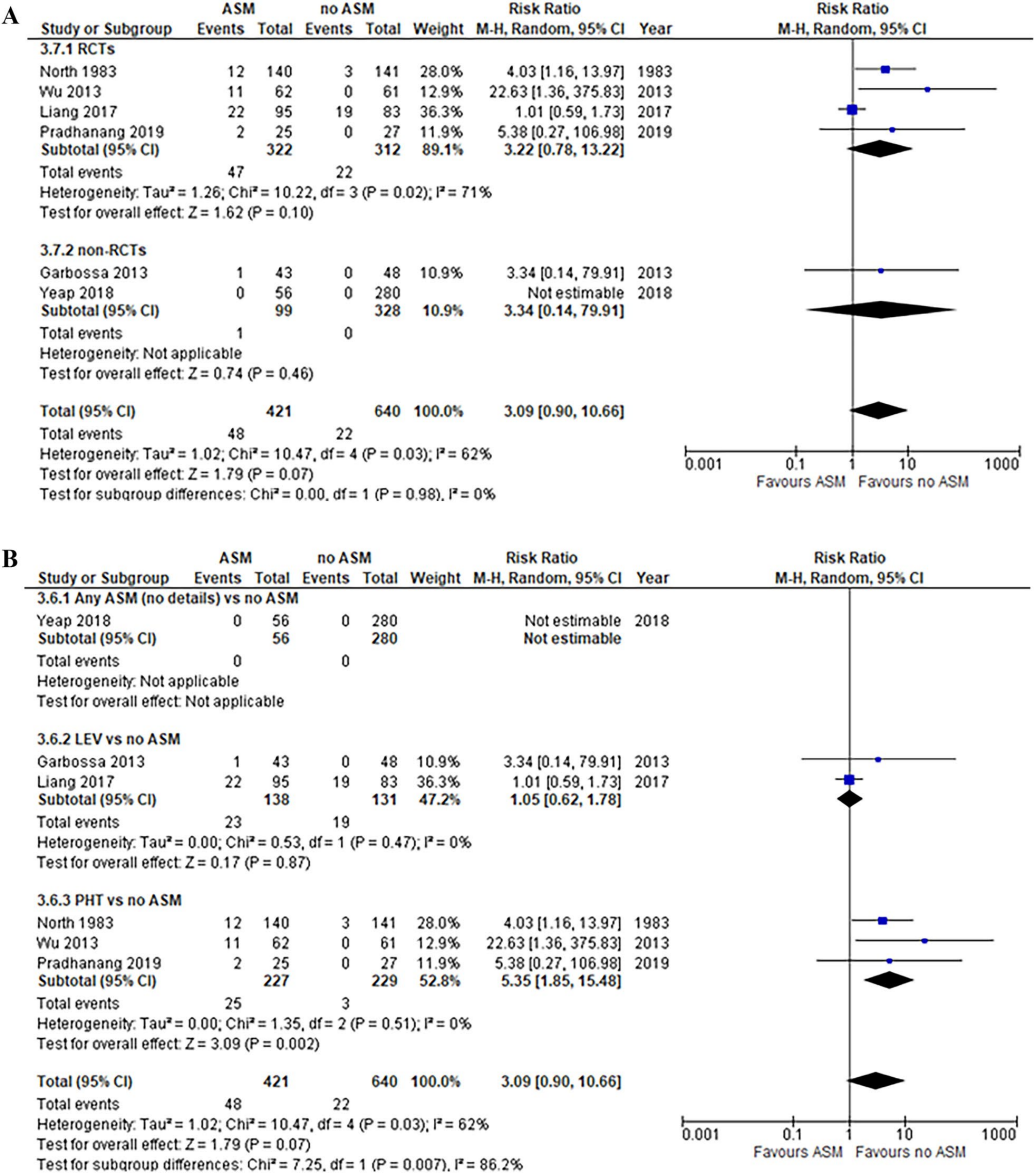
Meta-analysis (using a random-effects model) of adverse event outcomes among patients undergoing supratentorial neurosurgery stratified by **A** randomized versus nonrandomized study design and **B** ASM type

- c. Adverse event rates in ASM versus no ASM groups:

Six studies compared adverse events in patients treated with ASM versus no ASM [18, 20, 24, 25, 27, 29]. The adverse events reported in these articles included rash, involuntary movements, hirsutism, headache, discomfort in the face, thrombocytopenia, decreased level of consciousness, confusion, increased LFTs, nausea, vomiting, dry-itchy skin, ataxia, photophobia, aphasia, and anemia; however, these studies did not report on the occurrence of psychological or behavioral adverse events that can occur with LEV. The follow-up time reported in the trials varied but was up to 1 year of observation time. The relative risk of adverse events was higher in the pooled estimate of ASM-treated patients compared with nontreated patients; however, this did not reach statistical significance (RR 3.09, 95% CI 0.90–10.66, *I*^2^ = 62%, *p* = 0.07) (Fig. 4A and B). The pooled relative risk of adverse events in the treatment groups was significant in the three studies comparing PHT treatment with no ASM (RR 5.35, 95% CI 1.85–15.48 *I*^2^ = 0%, *p* = 0.002) [23, 24, 27]. In the two studies comparing treatment with LEV to controls, there was no difference in the risk ratio for adverse events (RR 1.05, 95% CI 0.62–1.78, *I*^2^ = 0%, *p* = 0.87) [20, 25].

**Fig. 5:**
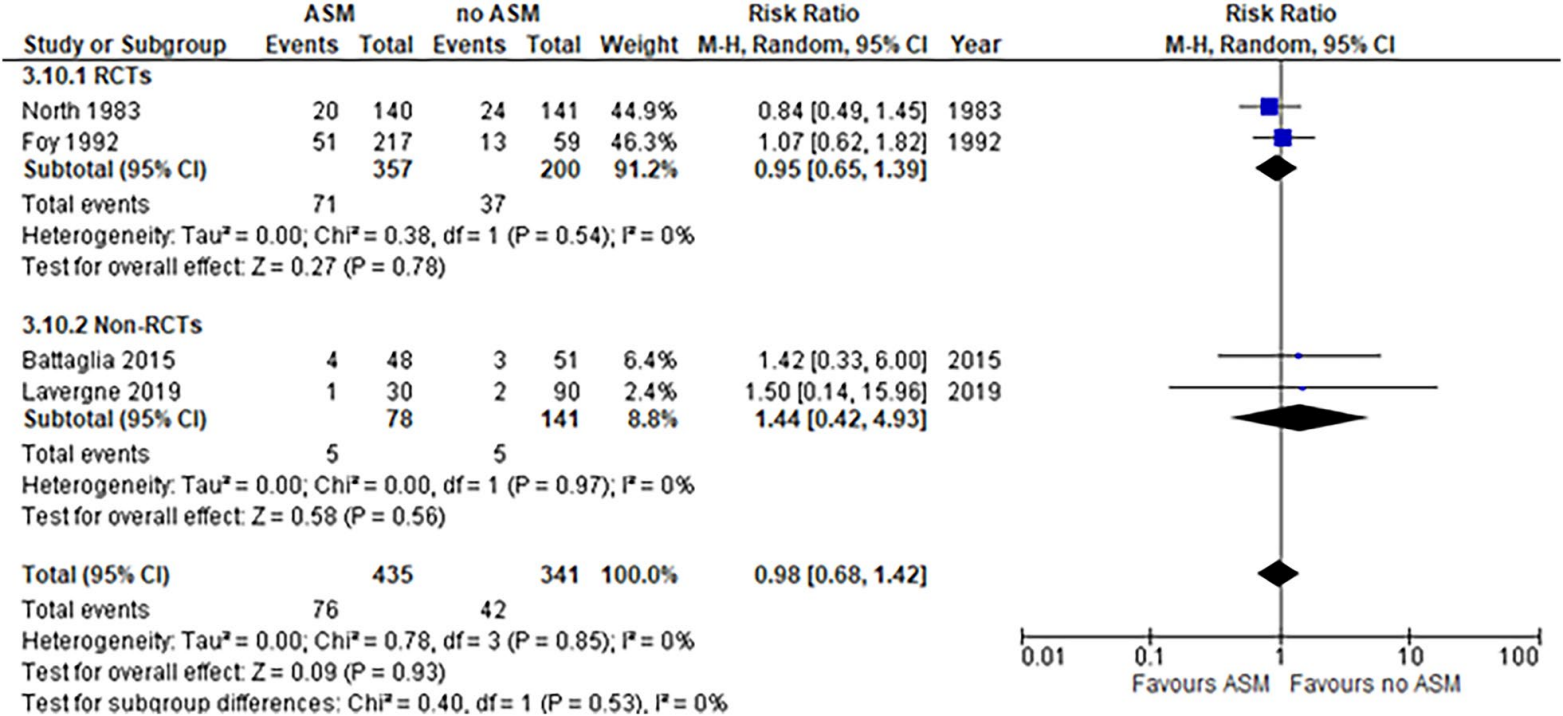
Meta-analysis (using a random-effects model) of mortality outcomes among patients undergoing supratentorial neurosurgery

- d. Mortality and functional outcomes in ASM versus no ASM groups:

Four studies (two RCT and two non-RCT) compared mortality in patients treated with ASM versus no ASM [19, 24, 26, 30]. These studies found no difference in mortality in patients treated with ASMs versus without ASMs (RR 1.26, 95% CI 0.78–2.05, *I*^2^ = 24%, *p* = 0.35) (Fig. 5).

Two studies reported functional outcomes data; however, the outcomes were too different to allow for a meta-analysis. One randomized controlled trial compared a good outcome of Modified Rankin Score (mRS) 0–1 at 6 months to mRS ≥ 2 [27]. This study found no difference in good outcome at 6 months for treatment with ASM versus no ASM (RR 0.99, 95% CI 0.75–1.04). Another study comparing the use of LEV for up to 24 weeks with no ASM found no differences in neuropsychological outcomes (Wechsler Intelligence Scale, full-scale memory quotient, and Barthel Index Activities of Daily Living) [25].

- e. Limitations in literature and future research needs:

First, there are several limitations in existing literature. The studies showing more adverse events in ASM-treated patients were those comparing PHT with no ASM [23, 24, 27]; additional large trials with LEV and other newer ASMs versus no ASMs are needed. These may add to the current body of knowledge implying newer ASMs may not have a higher rate of adverse events compared with no ASMs; however, it should be noted that behavioral and psychological adverse events by newer agents such as LEV may be underreported in these studies [20, 25].

Second, there was variability in the length of follow-up. For example, the North et al*.* study followed patients for up to 1 year for the development of seizures [24]. However, studies such as Lee et al. only followed patients for 3 days postoperatively [22]. This heterogeneity limits comparisons between studies. In addition, the observed variability in these outcomes likely contributes to the wide confidence intervals associated with the outcome estimates.

No studies used an electroencephalogram (EEG) to assess subclinical seizures, and therefore the outcome of seizure was based on clinical recognition of seizures. Since some studies show that up to 65% of post-neurosurgical seizures can be subclinical [31], there is a high probability for bias in outcome detection in current literature. It should be noted that limited evidence has evaluated unfavorable outcomes, such as decreased quality of life and epilepsy development, in patients undergoing craniotomy and experiencing subclinical seizures (i.e., only detectable with an EEG). However, in other disease states the occurrence of subclinical seizures detected by EEG has been associated with unfavorable outcomes such as delayed recovery, epilepsy, and decreased functional outcomes [32–34]. As such, it is difficult to estimate the effect of potentially undetected subclinical seizures on the outcomes evaluated in this guideline.

Blood levels of ASMs were not consistently reported. In the North et al*.* study, 81% of the patients in the PHT group had PHT levels in the therapeutic range during the study, and the average dose required to achieve a therapeutic level was 5.35 mg/kg/day (range 3.5–6.9 mg/kg/day) [24]. The study by Wu et al. reported that 100% of patients achieved a therapeutic PHT level (total level 10–20 mg/L) by postoperative day 7 [23]. In Lee et al. they described the use of a loading dose and adjustment of PHT dosage to total PHT levels of 10–20 mg/L but did not report PHT levels or the proportion of patients who achieved a therapeutic level [22]. In one study, LEV was dosed between 500 mg/day and 1500 mg/day [25] without blood level monitoring, and may have been underdosed compared with pharmacological data implying higher dosing requirements for critically ill patients that often have augmented renal clearance [13, 35].

In addition, there was substantial variability regarding indications for supratentorial neurosurgery, and the majority of studies were not randomized. When looking at the occurrence of short-term seizures, three of the studies comparing ASM with no ASM were randomized [22, 23, 36], five were not randomized [17–21], and clinical patient differences (including severity of underlying pathology) likely influenced which patients received ASM prophylaxis, introducing substantial bias to the evidence.

- f. Certainty of evidence:

There was substantial heterogeneity between studies and across subgroups for most of the outcomes of interest. The certainty of evidence assessment revealed serious concerns about residual confounding and publication bias (Table 3). Effect sizes for seizure prevention were trivial to small, and the imprecision of the estimates was high. When comparing ASM with no ASM for early seizure, only the subgroup of patients enrolled in an RCT who received PHT showed a favorable outcome, but this may be offset by the increased risk of adverse events seen in patients who received PHT. The exception to this low certainty was the subgroup analysis combining the two randomized controlled trials examining mortality, which found high certainty of there being no difference in mortality between treatment with and without prophylactic ASMs in patients undergoing supratentorial neurosurgery. Overall, there was low certainty of evidence for the question of prophylactic ASM use in patients undergoing supratentorial neurosurgery (Table 4).

- g. Recommendation:

**Table 3:** PICO 1—certainty assessment tables for prophylactic use of ASM versus no ASM for supratentorial neurosurgery

Certainty assessment							No. of patients		Effect		Certainty	Importance
No. of studies	Study design	Risk of bias	Inconsistency	Indirectness	Imprecision	Other considerations	An anti-seizure medication	No anti-seizure medication	Relative (95% CI)	Absolute (95% CI)		
*Prevention of short-term seizures*												
6 [17, 18, 20, 25, 30]	Nonrandomized studies	Serious^a^	Serious^b^	Not serious	Serious^b^	All plausible residual confounding would reduce the demonstrated effect	13/306 (4.2%)	53/661 (8.0%)	RR 0.61 (0.12 to 3.03)	31 fewer per 1000 (from 71 fewer to 163 more)	⨁⨁◯◯ Low	CRITICAL
*Prevention of short-term seizures*												
3 [29, 36]	Randomized trials	Serious^b^	Serious^b^	Not serious	Serious^b^	Publication bias strongly suspected, all plausible residual confounding would reduce the demonstrated effect^b^	9/391 (2.3%)	31/387 (8.0%)	RR 0.29 (0.14 to 0.61)	57 fewer per 1000 (from 69 to 31 fewer)	⨁◯◯◯ Very low	CRITICAL
*Prevention of long-term seizures*												
2 [18, 20]	Nonrandomized studies	Serious^a^	Serious^b^	Not serious^b^	Serious^b^	All plausible residual confounding would reduce the demonstrated effect	23/99 (23.2%)	57/328 (17.4%)	RR 1.35 (0.73 to 2.46)	61 more per 1000 (from 47 fewer to 254 more)	⨁◯◯◯ Very low	
*Prevention of long-term seizures*												
5 [25, 29, 36, 45]	Randomized trials	Serious^b^	Serious^b^	Not serious	Serious^b^	Publication bias strongly suspected^b,c^	104/541 (19.2%)	51/388 (13.1%)	RR 0.93 (0.62 to 1.40)	9 fewer per 1000 (from 50 fewer to 53 more)	⨁◯◯◯ Very low	
*Adverse events*												
2 [18, 20]	Nonrandomized studies	Serious^a,b^	Serious^b^	Not serious	Serious^b^	All plausible residual confounding would reduce the demonstrated effect	1/99 (1.0%)	0/328 (0.0%)	RR 3.34 (0.14 to 79.91)	0 fewer per 1000 (from 0 to 0 fewer)	⨁◯◯◯ Very low	
*Adverse events*												
4 [25, 29, 36]	Randomized trials	Serious^b,c^	Serious^b^	Not serious	Serious^b^	Publication bias strongly suspected^c^	47/322 (14.6%)	22/312 (7.1%)	RR 3.22 (0.78 to 13.22)	157 more per 1000 (from 16 fewer to 862 more)	⨁◯◯◯ Very low	
*Mortality*												
2 [26, 36]	Randomized trials	Not serious	Not serious	Not serious	Not serious	Publication bias strongly suspected, all plausible residual confounding would reduce the demonstrated effect^c^	41/191 (21.5%)	37/200 (18.5%)	RR 1.24 (0.57 to 2.73)	44 more per 1000 (from 80 fewer to 320 more)	⨁⨁⨁⨁ High	
*Mortality*												
2 [19, 21]	Nonrandomized studies	Serious	Not serious	Not serious	Not serious	None	5/78 (6.4%)	5/141 (3.5%)	RR 1.44 (0.42 to 4.93)	16 more per 1000 (from 21 fewer to 139 more)	⨁⨁⨁◯ Moderate	

^a^Retrospective study

^b^Small number of studies

^c^Inconsistent doses or no monitoring of drug concentrations

^d^Signifi cant heterogeneity

**Table 4:** PICO 1 summary of judgments for conditional recommendation for either ASM (intervention) or no ASM (comparison)

	Judgement						
Problem	No	Probably no	Probably yes	Yes		Varies	Do not know
Desirable effects	Trivial	Small	Moderate	Large		Varies	Do not know
UNDESIRABLE EFFECTS	Large	Moderate	Small	Trivial		Varies	Do not know
Certainty of evidence	Very low	Low	Moderate	High			No included studies
Values	Important uncertainty or variability	Possibly important uncertainty or variability	Probably no important uncertainty or variability	No important uncertainty or variability			
Balance of effects	Favors the comparison	Probably favors the comparison	Does not favor either the intervention or the comparison	Probably favors the intervention	Favors the intervention	Varies	Do not know
Acceptability	No	Probably no	Probably yes	Yes		Varies	Do not know
Feasibility	No	Probably no	Probably yes	Yes		Varies	Do not know

*We suggest that clinicians may choose to use ASM or not use ASM for seizure prevention in patients undergoing supratentorial neurosurgery, as the balance of benefits and harms is uncertain (conditional recommendation, low certainty of evidence).*

Justification: Across all outcomes of interest, when pooled, we did not detect a significant positive effect of prophylactic ASM compared with no ASM treatment for the outcomes of early seizure, late seizure, or mortality. Though the subgroup of patients enrolled in an RCT who received PHT had a lower risk of developing early seizures, PHT was also associated with a significantly higher risk of adverse events compared with no ASM. However, it should be noted that the behavioral adverse events associated with LEV were likely underreported in current literature.

*PICO 2: If an anti-seizure medication is used, should LEV or PHT be preferentially used for supratentorial neurosurgery in patients with no history of clinical or electrographic seizures?*

**Fig. 6:**
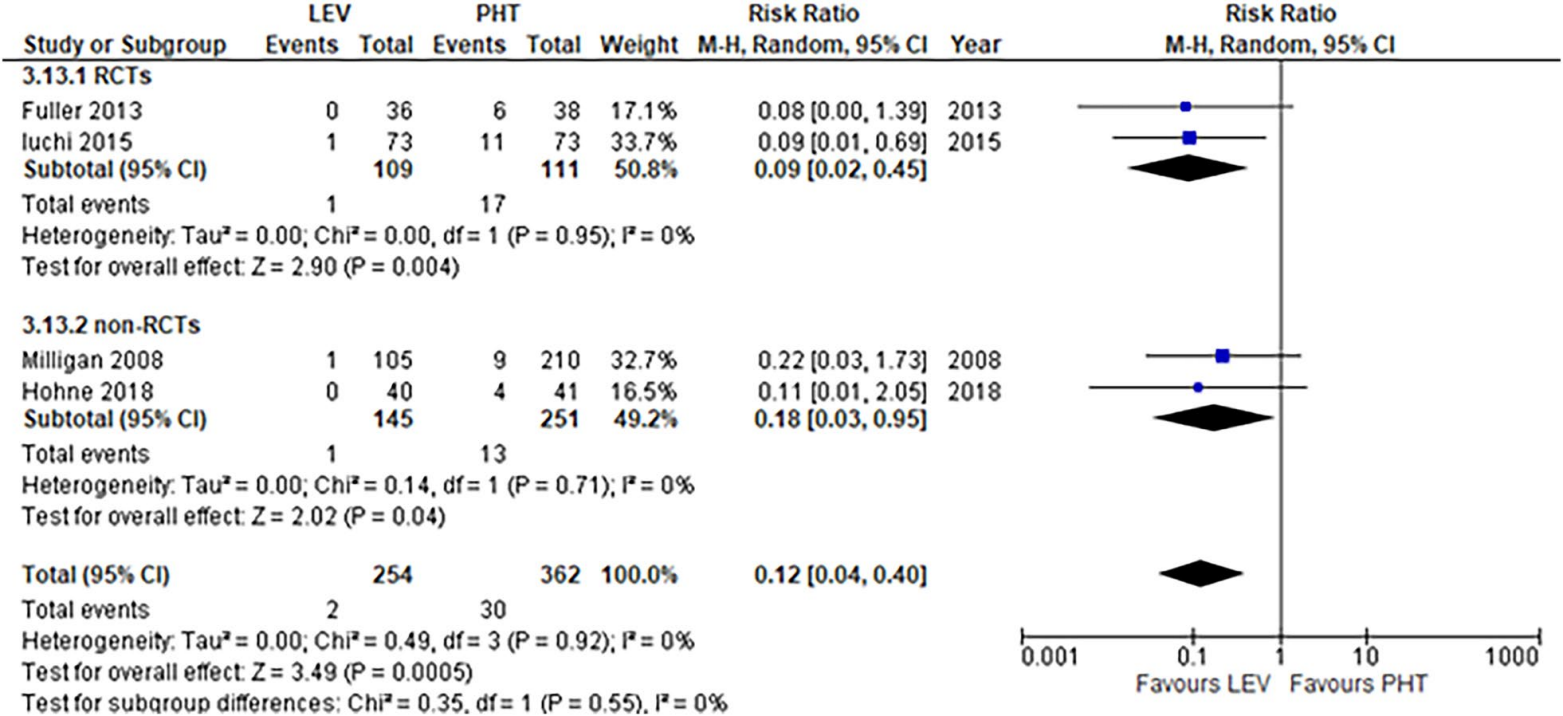
Meta-analysis of early seizure outcome in patients treated with prophylactic LEV compared with PHT. The overall effect, RCT and non-RCT subgroups show significant differences between LEV and PHT, favoring LEV

- a. To prevent early seizures (≤ 14 days from neurosurgery or during hospitalization) using LEV vs. PHT:

Four studies, consisting of two randomized controlled trials (RCTs) [37, 38], and two nonrandomized trials [39, 40] with a total of 616 patients, were included in the meta-analysis. All studies reported clinical seizures and did not utilize EEG for seizure detection. The dosages of both LEV and PHT varied across these trials. The most common dosages of LEV and PHT were 500 mg twice daily and 100 mg three times daily, respectively. Only one study reported LEV levels, but the levels were non-steady-state, post-load levels (mean 9.4 ± 3.7 μg/mL) [38]. As it relates to PHT levels, two of the four studies reported levels [38, 39]. In Iuchi et al., the authors reported a non-steady-state, post-load average level of 9.9 ± 2.9 μg/mL [38]. In the Milligan et al. study, the median steady-state total PHT levels of 11 μg/mL (range 1–27 μg/mL) [39]. Overall, there was a significant difference favoring LEV over PHT for early seizure prevention (RR 0.12, 95% CI 0.04–0.40, *I*^2^ = 0%, *p* = 0.0005). There was no heterogeneity between the studies (*I*^2^ = 0%, *p* = 0.92) or in subgroups of RCTs versus non-RCTs (*I*^2^ = 0%, *p* = 0.55) (Fig. 6).

**Fig. 7:**
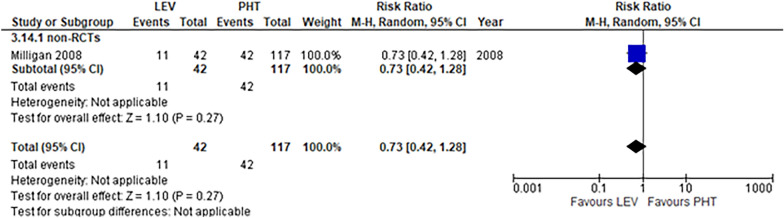
Meta-analysis of late-seizure outcomes in patients treated with prophylactic LEV compared with PHT. Only one RCT reported data, no significant difference is seen between LEV and PHT

**Fig. 8:**
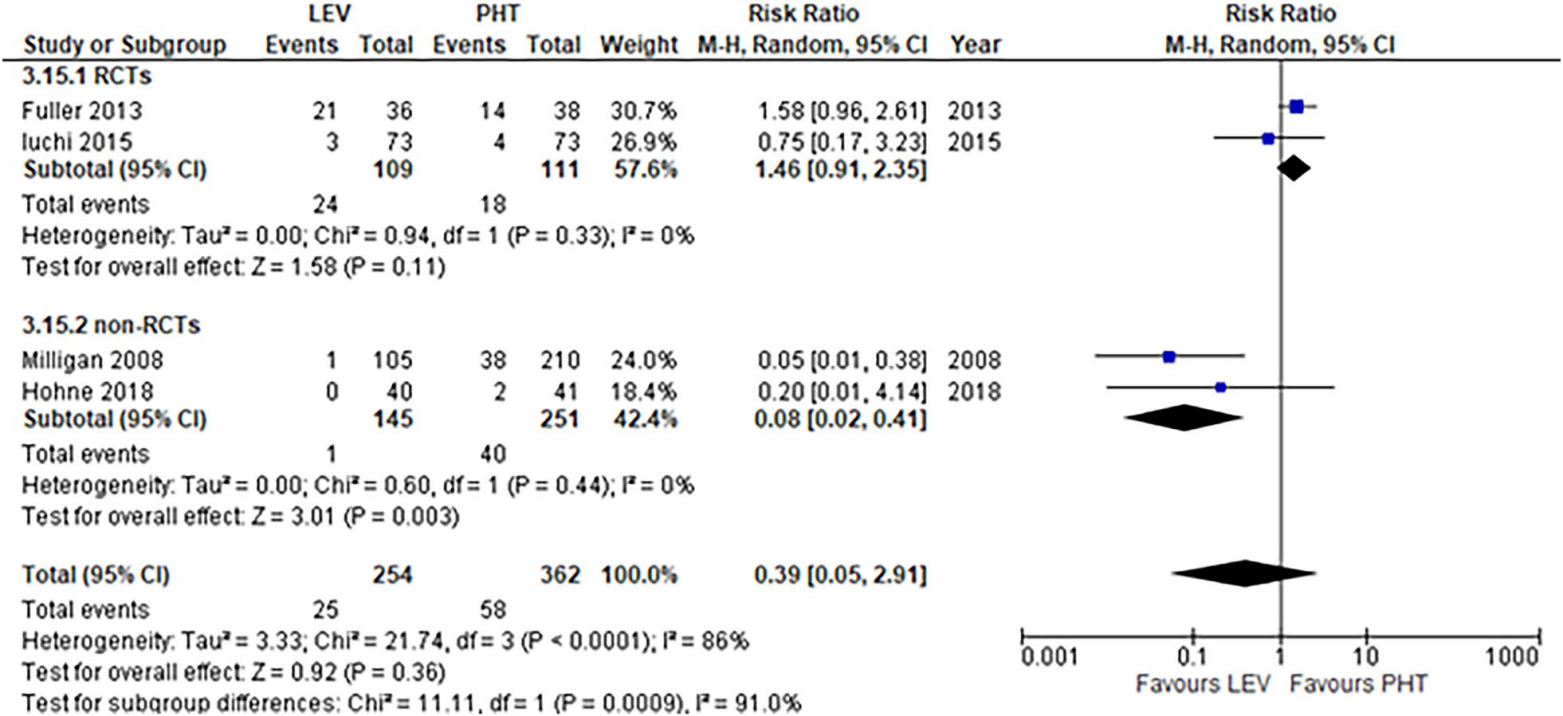
Meta-analysis of adverse events in patients treated with prophylactic LEV compared with PHT. One non-RCT shows a significant difference favoring leviteracetam. Significant heterogneity between studies, as well between subgroups, is seen

In one of the RCTs, a separate analysis was conducted for 110 of the 146 patients who never had preoperative seizures. The odds of having early seizures in the LEV group reached statistical significance (OR 8.016, 95% CI 1.42–154.19, *p* = 0.015). Of note, these investigators measured PHT serum levels, which were found to be at the lower end of the therapeutic range. The difference between serum levels in patients with or without postoperative seizure was not statistically significant (*p* = 0.72) [38].

b. To prevent late seizures (> 14 days from neurosurgery or post-hospitalization) using LEV or PHT:

One observational study reported results for late seizures in patients undergoing supratentorial neurosurgery [39]. Though 315 patients were included in the initial analysis, only 159 were followed for the late-seizure outcome. The median follow-up time in the study for the LEV and PHT groups was 5 months (range 0.25–52 months) and 12 months (range 0.25–60 months), respectively. A total of 11 late-seizure events among 42 patients were noted for LEV (26%) compared with 42 among 117 patients for PHT (36%, RR 0.73, 95% CI 0.42–1.28, *p* = 0.27) (Fig. 7). It should be noted that the authors of this study did not statistically control for the differences in observation time for the two groups. Serum PHT levels were monitored in this study; however, there was no statistically significant difference in serum levels between those who did and those who did not experience postoperative seizures (*p* = 0.72). No LEV levels were monitored in the study.

c. Adverse event rates of LEV vs. PHT:

Four studies evaluated the incidence of adverse events, including two randomized controlled trials [37, 38] and two nonrandomized trials [39, 40], with a total of 616 patients. Reported adverse events for LEV included rash, delirium, headache, itching, liver dysfunction, and visual hallucinations. Reported adverse events for PHT included anaphylaxis, severe allergic reaction, thrombophlebitis, rash, ataxia, nausea, liver dysfunction, hyponatremia, atrial fibrillation, fever, cytopenia, rhabdomyolysis, cognitive change, and tremor. The follow-up time for reporting adverse events varied with two studies following patients for 7 days [38, 40], one for up to 90 days [37], and one for up to 12 months of follow-up [39]. Significant heterogeneity was noted among the studies (*I*^2^ = 86%, *p* < 0.001) and between the RCT and non-RCT subgroups (*I*^2^ = 91%, *p* < 0.001). Overall, when comparing LEV and PHT there was no significant difference in the risk of adverse events (RR 0.39, 95% CI 0.05–2.91, *I*^2^ = 86%, *p* = 0.36) (Fig. 8). A separate analysis was performed for one RCT regarding side effects requiring discontinuation of the ASM [37]. No significant difference among LEV and PHT groups were noted (RR 0.53, 95% CI 0.05–5.57, *p* = 0.60).

d. Comparison of other anti-seizure medications:

Two RCTs [26, 41] compared PHT and carbamazepine. Late-seizure incidence was similar in both groups (RR 0.91, 95% CI 0.57–1.45 and RR 0.81, 95% CI 0.33–2.01, respectively; *p* = 0.57). In Foy et al. [26] mortality rates were not significantly different among the PHT and carbamazepine groups (RR 1.52, CI 0.73–3.15, *p* = 0.27). In Shaw et al. [32], there was a trend toward an increased rate of adverse events in patients who received carbamazepine, but this did not reach statistical significance (RR 0.65, 95% CI 0.23–1.85, *p* = 0.42).

One non-RCT [42] compared LEV and valproic acid. Early-seizure incidence was not significantly different between the groups (RR 1.21, 95% CI 0.42–3.49, *p* = 0.73), but the adverse event rate was significantly higher for valproic acid compared with LEV (RR 0.37, 95% CI 0.15–0.86, *p* = 0.02).

e. Limitations in literature and future research needs:

In at least one nonrandomized study [39] there was significant loss to follow-up over time, introducing potential for outcome bias. There was also variability among studies as to the adherence to serum PHT monitoring. Fuller et al. [37] did not monitor serum PHT, perhaps contributing to the benefit shown to LEV for reducing postoperative seizures. However, in the studies that did monitor these levels in some/many patients, there appeared to be no statistical evidence that maintaining the serum PHT level impacted the incidence of seizure [38, 39]. One study [38] did address the issue of subject ASM noncompliance, which did not result in a statistically significant incidence of postoperative seizures, but left open the question of the potential influence of such noncompliance.

In addition, there was significant heterogeneity in the indication for craniotomy. The risk for postoperative seizure is dependent on surgical indication, lesion location, lesion pathology, and type of surgery [17, 28]. As the studies included many different indications, it is difficult to determine which craniotomy patients would be at highest risk and potentially benefit from either LEV or PHT.

Another potential limitation in the comparison of LEV and PHT is the lack of consistent level monitoring. Only one of the included studies evaluated LEV levels [38]. While there is no universal agreement on the therapeutic range for LEV, there is the potential for underdosing patients when the most commonly used dose was 500 mg twice daily. Two of the included studies did describe the use of PHT level monitoring for adjustment to dosage [38, 39]. The lack of consistent drug level monitoring and low dosages of LEV may contribute to potential bias toward one treatment. As with PICO 1 (above), studies relied on clinical seizure detection only and, thus, electrographic seizures may have been missed.

f. Certainty of evidence:

In assessing early seizure incidence and adverse events, publication bias was strongly suspected in the RCTs. For early- and late-seizure incidence and adverse events, the observational studies (non-RCTs) were considered to have residual confounding factors that would reduce the demonstrated effect. For all of the studies analyzed, the certainty of the evidence was very low, with a serious risk of bias overall (Table 5).

g. Recommendation PICO 2:

**Table 5:** PICO 2—certainty assessment tables for prophylactic LEV vs. PHT after supratentorial neurosurgery

Certainty assessment							No. of patients		Effect		Certainty	Importance
No. of studies	Study design	Risk of bias	Inconsistency	Indirectness	Imprecision	Other considerations	Levetiracetam	Phenytoin	Relative (95% CI)	Absolute (95% CI)		
*Early seizure*												
2 [39]	Nonrandomized studies	Serious^a^	Not serious	Serious	Not serious	All plausible residual confounding would reduce the demonstrated effect	1/145 (0.7%)	13/251 (5.2%)	RR 0.18 (0.03 to 0.95)	42 fewer per 1000 (from 50 to 3 fewer)	⨁◯◯◯ Very low	
*Early seizure*												
2 [37, 38]	Randomized trials	Serious^b^	Not serious	Serious^c^	Not serious	Publication bias strongly suspected^b^	1/109 (0.9%)	17/111 (15.3%)	RR 0.09 (0.02 to 0.45)	139 fewer per 1000 (from 150 to 84 fewer)	⨁◯◯◯ Very low	
*Adverse events*												
2 [40, 40]	Nonrandomized studies	Serious^a^	Not serious	Not serious	Serious^d^	All plausible residual confounding would reduce the demonstrated effect	1/145 (0.7%)	40/251 (15.9%)	RR 0.08 (0.02 to 0.41)	147 fewer per 1000 (from 156 to 94 fewer)	⨁◯◯◯ Very low	
*Adverse events*												
2 [37, 38]	Randomized trials	Serious^b,c^	Not serious	Not serious	Serious^d^	Publication bias strongly suspected^b^	24/109 (22.0%)	18/111 (16.2%)	RR 1.46 (0.91 to 2.35)	75 more per 1000 (from 15 fewer to 219 more)	⨁◯◯◯ Very low	
*Late seizure*												
1 [39]	Nonrandomized studies	Serious^a,b^	Serious^b^	Not serious	Not serious	All plausible residual confounding would reduce the demonstrated effect	11/42 (26.2%)	42/117 (35.9%)	RR 0.73 (0.42 to 1.28)	97 fewer per 1000 (from 208 fewer to 101 more)	⨁◯◯◯ Very low	

^a^Retrospective study

^b^Small number of studies

^c^Inconsistent doses or no monitoring of drug concentrations

^d^Signifi cant heterogeneity

*When prophylactic ASM is used for supratentorial craniotomy, we suggest LEV over PHT (conditional recommendation, very-low certainty of evidence)* (Table 6).

**Table 6:** Summary of judgments for conditional recommendation for LEV (intervention) over PHT (comparator)

	Judgement						
Problem	No	Probably no	Probably yes	Yes		Varies	Do not know
Desirable Effects	Trivial	Small	Moderate	Large		Varies	Do not know
Undesirable Effects	Large	Moderate	Small	Trivial		Varies	Do not know
Certainty of evidence	Very low	Low	Moderate	High			No included studies
Values	Important uncertainty or variability	Possibly important uncertainty or variability	Probably no important uncertainty or variability	No important uncertainty or variability			
Balance of effects	Favors the comparison	Probably favors the comparison	Does not favor either the intervention or the comparison	Probably favors the intervention	Favors the intervention	Varies	Do not know
Acceptability	No	Probably no	Probably yes	Yes		Varies	Do not know
Feasibility	No	Probably no	Probably yes	Yes		Varies	Do not know

Justification: Owing to ease of use and a perceived decrease in adverse events as compared with other ASDs, LEV use has increased in the last several years as a preferred agent for prophylaxis for supratentorial craniotomies for various pathologies, including vascular lesions, tumors, and trauma, rendering this question a high priority. The primary outcomes analyzed for this PICO were early- and late-seizures and adverse events. LEV is significantly better than PHT in early postoperative seizure prevention. In one non-RCT, LEV was favored owing to fewer adverse events and the overall point estimate for adverse events favored LEV. LEV was also favored over valproic acid for adverse event incidence. Although the certainty of the evidence is very low, the desirable effects of LEV (seizure prevention) are moderate compared with trivial undesirable effects (adverse events), the balance of which favors LEV.

*PICO 3: If an anti-seizure medication is used, should a long (*> *7 days) versus short (*≤ *7 days) duration of prophylaxis be used for supratentorial neurosurgery in patients with no history of clinical or electrographic seizures?*

- To prevent early seizures (≤ 14 days from neurosurgery or during hospitalization) using short versus long durations of ASM:

Only one study met our definition of long- and short-term therapy and evaluated the effect of duration of therapy on early seizures [43]. Because only one study fits our definition of duration of therapy, we did not conduct a meta-analysis for this outcome. Rahman et al. describe a randomized controlled trial where postoperative supratentorial tumor resection patients were randomized to either 1 week of LEV (*N* = 40) or 6 months of LEV (*N* = 41). There was one seizure in each of the groups that occurred during the first week of therapy. It should be noted that patients received LEV 1000 mg extended release daily or 500 mg twice daily for the duration of the trial.

b. To prevent late seizures (> 14 days from neurosurgery or post-hospitalization):

Two studies evaluated duration of therapy on late seizures. Foy et al. evaluated the use of carbamazepine or PHT for 6 months or 24 months. Patients with aneurysm, arteriovenous malformation, spontaneous hematoma, abscess, meningioma, and benign tumors were included. A total of 43 of 105 (40.9%) patients in the short-term group and 36 of 112 (32.1%) patients in the long-term group developed seizures. Overall, there was no difference in the occurrence of late seizures as patients were followed for up to 24 months (*p* = 0.33) [26]. The other trial was a retrospective cohort study that compared short-term use (less than 1 month) (*N* = 216) to long-term use (3–6 months) (*N* = 165) of PHT following craniotomy for aneurysmal clipping. The authors did not report how long the patients were followed; however, they defined late seizures as a seizure that developed after hospital discharge. In those patients without perioperative seizures, there was no difference in late seizures [44].

c. Adverse event rates with short versus long durations of ASM:

One study evaluated the effect of LEV duration on adverse events [43]. The authors of the study reported 30 serious and “other” adverse events that ranged in frequency from 1.3% to 15.3%. Overall, the most common adverse events were headache, fatigue, nausea, and confusion. While the authors did not do a formal statistical analysis of the incidence of adverse events, the incidence of adverse events did not appear to increase with the duration of LEV. However, when comparing ASM with no ASM use, there was a trend toward more adverse events. Hence, longer exposure to ASM would conceivably increase the risk of accruing adverse events over time.

d. Limitations in literature and future research needs:

In our literature search, only one randomized controlled trial met our definition of short- and long-term therapy. While two other trials, a randomized controlled trial and a retrospective cohort study, did evaluate the effect of ASM duration, both the short- and long-term therapy arms would be considered long-term therapy by our definition. Owing to the sparsity of evidence available to evaluate this PICO question, it is difficult to draw direct conclusions concerning the effect of ASM duration on the development of early seizures, late seizures, and adverse events. As such, the evidence supporting this PICO is generally indirect and this question represents a significant area for future research.

e. Certainty of evidence:

Overall, the certainty of evidence related to this is very low. There was a very high risk of bias, inconsistency, indirectness, imprecision, and other considerations. In addition, the lack of published trials implies a risk of publication bias (Table 7).

f. Recommendation PICO 3:

**Table 7:** PICO 3—certainty assessment tables for duration of prophylactic ASD after supratentorial neurosurgery

Certainty assessment							No. of patients		Effect		Certainty	Importance
No. of studies	Study design	Risk of bias	Inconsistency	Indirectness	Imprecision	Other considerations	A long duration of anti-seizure medication	A short duration of anti-seizure medication	Relative (95% CI)	Absolute (95% CI)		
*Late seizure*												
1	Nonrandomized studies	Extremely serious	Serious	Serious	Serious	Publication bias strongly suspected, all plausible residual confounding would reduce the demonstrated effect	6/160 (3.8%)	9/238 (3.8%)	Not estimable		⨁◯◯◯ Very low	
*Late seizure*												
2	Randomized trials	Serious	Serious	Not serious	Serious	None	37/153 (24.2%)	44/145 (30.3%)	Not estimable		⨁◯◯◯ Very low	

CI, confidence interval; RR, risk ratio

*If a prophylactic ASM is used for supratentorial craniotomy, we suggest a short duration (*≤ *7 days) vs. a longer duration (*> *7 days) of use (conditional recommendation, very-low certainty of evidence)* (Table 8).

**Table 8:** Summary of judgments for conditional recommendation for short- or long-term therapy

	Judgement						
Problem	No	Probably no	Probably yes	Yes		Varies	Do not know
Desirable effects	Trivial	Small	Moderate	Large		Varies	Do not know
Undesirable Effects	Large	Moderate	Small	Trivial		Varies	Do not know
Certainty of evidence	Very low	Low	Moderate	High			No included studies
Values	Important uncertainty or variability	Possibly important uncertainty or variability	Probably no important uncertainty or variability	No important uncertainty or variability			
Balance Of Effects	Favors the comparison	Probably favors the comparison	Does not favor either the intervention or the comparison	Probably favors the intervention	Favors the intervention	Varies	Do not know
Acceptability	No	Probably no	Probably yes	Yes		Varies	Do not know
Feasibility	No	Probably no	Probably yes	Yes		Varies	Do not know

Justification: Though there is significant paucity of evidence related to this question, the three studies that addressed the duration of ASM use did not demonstrate a benefit to prolonged ASM use, and longer exposure to ASM would conceivably increase the risk of adverse events. As such, the group felt that if an ASM were going to be used for prophylaxis, it would be most appropriate to limit the duration.

## Discussion

The authors of this guideline have evaluated available literature and came to consensus concerning the use of ASM in patients who have undergone supratentorial neurosurgery. With the goal of providing practical guidance, the committee developed the following “In Our Practice” expert opinion statements for each PICO question on the basis of group discussion and consensus.

General Recommendations: Because the balance of potential benefits and harms of prophylactic ASM use remains uncertain, and because seizure risk varies on the basis of individual patient factors, decisions about whether to initiate ASM prophylaxis, which agent to select, and how long to continue therapy should be revisited throughout the inpatient course of patients undergoing supratentorial surgery. When appropriate, incorporating these recommendations into care pathways, checklists, and order sets can help support consistent decision-making and reinforce the underlying clinical equipoise. In addition, the authors encourage institutions to track local data on seizure incidence, ASM prophylaxis use, and adverse events. These data are invaluable to informing decisions about the care of patients at individual institutions. Although this guideline does not evaluate the outcomes of adding ASM-related decision points to standardized order sets, this approach reflects common practice within our institutions.

PICO 1: use of ASM or no ASM in our practice: On the basis of RCTs alone, there was a significant benefit for early seizure prevention using ASM; however, this effect was nonsignificant in the meta-analysis that included significantly heterogeneous non-RCTs with inferior methodological design. It should be noted that the randomized controlled trials in this evaluation were graded to be of low-quality evidence with significant bias and flaws. This may have also contributed to the lack of statistically significant results. Furthermore, the risk of adverse events related to the short-term use of ASM is relatively low when utilizing newer generation ASM as prophylaxis. Indeed, meta-analyses of adverse events in studies utilizing LEV did not show any increased risk when compared with no ASM. In addition, we acknowledge that there may be differences in seizure risk based on lesion location and indication for supratentorial surgery; however, the existing literature is sparse, heterogeneous, and rarely stratified by underlying surgical indication. These potential differences in seizure risk can influence the decision to utilize ASM prophylaxis. Thus, out of caution, it is our usual practice to use prophylactic ASMs in the majority of patients undergoing supratentorial neurosurgery.

PICO 2: LEV vs. PHT in our practice: In meta-analyses, LEV was associated with a reduced risk of early seizures compared with PHT; however, interestingly, we did not observe the same difference when evaluating adverse events. The inability to detect a difference in adverse events in the LEV group could be attributed to the significant heterogeneity and small sample sizes of the included trials. In addition, many of the included studies did not focus on the potential for behavioral adverse events that may be associated with LEV [45]. The authors came to consensus that in our practice, LEV has become the predominant first-line ASM for prophylaxis in patients undergoing supratentorial neurosurgery owing to its low toxicity profile, limited drug interactions, and lack of significant albumin binding, which can cause substantial fluctuations in drug levels. While LEV has gained wider usage among providers for these reasons, certain side effects of LEV, especially in older patients, such as drowsiness, delirium, dizziness, and pancytopenia, as well as limitations in patients with renal insufficiency/failure have also increased the use of newer, alternative agents (e.g., lacosamide, clobazam, and brivaracetam). It should be noted that the LEV dose should be reduced in patients with varying degrees of renal disfunction as measured by creatinine clearance. In patients with a creatinine clearance of less than 30 mL/min the dose should not exceed 500 mg every 12h. That should be further reduced to no greater than 500 mg every 24h in those patients with a creatinine clearance of less than 15 mL/min. Despite the potential problems with LEV, it is our general practice to use LEV if employing seizure prophylaxis. Randomized controlled trials are needed to further compare the prophylactic effects of LEV with these newer ASMs as monotherapy. In addition, it should be noted that we did not observe a difference in prevention of seizures with LEV when compared with no treatment (PICO 1), but we did observe a significant reduction in early seizures when LEV was compared with PHT. This apparent paradoxical difference in outcome may be attributable to many different variables including but not limited to differences in study design, sample size, dosing of medications, differences in population, and surgery etiology.

PICO3: Duration of ASM in our practice: The authors found very limited direct evidence for or against the use of short term ASM prophylaxis. There is variability amongst practitioners regarding the duration of ASM prophylaxis in the multiple conditions requiring supratentorial surgical interventions, such as tumors, trauma, infection, and vascular issues. Owing to concerns about potential adverse events with prolonged ASM use, the results of this meta-analysis may guide practitioners toward shorter therapy duration. Thus, in our practice, owing to concerns about potential adverse events with prolonged ASM use, the authors will utilize short-term ASM prophylaxis if ASM prophylaxis is to be used in patients who have undergone supratentorial neurosurgery.

## Conclusions

Overall, owing to a lack of evidence, the authors could not recommend for or against prophylactic ASM use for patients undergoing supratentorial neurosurgery, but suggest that if an ASM is to be used, LEV is preferred over PHT, and administered for a short duration of therapy. Because of the heterogeneous nature of supratentorial surgery, there is the potential for varying degrees of seizure risk based on patient specific characteristics such as lesion location, underlying indication, EEG findings, comorbidities, and others. As such, future research in the use of ASM for prophylaxis should focus on patient-specific characteristics and risk stratification.

## Supplementary Information

Below is the link to the electronic supplementary material.

Supplementary file1 (PDF 1774 KB)

## References

[1] Greenhalgh J, Weston J, Dundar Y, Nevitt SJ, Marson AG. Antiepileptic drugs as prophylaxis for postcraniotomy seizures. Cochrane Database Syst Revi. 2018;5:007286. 10.1002/14651858.CD007286.pub4 PMC6494638 29791030

[2] Englot DJ, Magill ST, Han SJ, et al. Seizures in supratentorial meningioma: a systematic review and meta-analysis. J Neurosurg. 2016;124(6):1552–61. 26636386 10.3171/2015.4.JNS142742 PMC4889504

[3] Sun B, Lu W, Yu W, Tian Y, Wang P. Prevalence and risk factors of early postoperative seizures in patients with glioma: a protocol for meta-analysis and systematic review. PLoS ONE. 2024;19(4):e0301443. 38574171 10.1371/journal.pone.0301443 PMC10994364

[4] GRADE. [cited 2022; Available from: https://www.gradeworkinggroup.org/.

[5] Schunemann, H.J.B., J.; Guyatt, G.; Oxman, A. *GRADE Handbook*. 2022; Available from: https://gdt.gradepro.org/app/handbook/handbook.html.

[6] *GRADE Guidelines Workshop + focus on implementation*. [cited 2022; Available from: https://gradeconf.org/2022-11/.

[7] Guyatt GH, Oxman AD, Kunz R, et al. Grade guidelines: 2 Framing the question and deciding on important outcomes. J Clin Epidemiol. 2011;64(4):395–400. 21194891 10.1016/j.jclinepi.2010.09.012

[8] van Swieten JC, Koudstaal PJ, Visser MC, Schouten, H.J.van Gijn, J. Interobserver agreement for the assessment of handicap in stroke patients. Stroke. 1988;19(5):604–7. 3363593 10.1161/01.str.19.5.604

[9] Jennett B, Bond M. Assessment of outcome after severe brain damage. Lancet. 1975;1(7905):480–4. 46957 10.1016/s0140-6736(75)92830-5

[10] Giraldi L, Vinslov Hansen J, Wohlfahrt J, et al. Postoperative de novo epilepsy after craniotomy: a nationwide register-based cohort study. J Neurol Neurosurg Psychiatry. 2022;93(4):436–44. 34845003 10.1136/jnnp-2021-326968 PMC8921591

[11] Streng ML, Krook-Magnuson E. The cerebellum and epilepsy. Epilepsy Behav. 2021;121(Pt B):106909. 32035793 10.1016/j.yebeh.2020.106909 PMC7415499

[12] Frontera JA, Rayi A, Tesoro E, et al. Guidelines for seizure prophylaxis in patients hospitalized with nontraumatic intracerebral hemorrhage: a clinical practice guideline for health care professionals from the Neurocritical Care Society. Neurocrit Care. 2025;42(1):1–21. 39707127 10.1007/s12028-024-02183-z

[13] Frontera JA, Gilmore EJ, Johnson EL, et al. Guidelines for seizure prophylaxis in adults hospitalized with moderate-severe traumatic brain injury: a clinical practice guideline for health care professionals from the Neurocritical Care Society. Neurocrit Care. 2024;40(3):819–44. 38316735 10.1007/s12028-023-01907-x

[14] Higgins, J.P.S., J; Page, MJ; Elbers, RG; Sterne, JAC, (2022) *Assessing risk of bias in a randomized trial*, in *Cochrane Handbook for Systematic Reviews of Interventions version 6.3*, T.J. Higgins JPT, Chandler J, Cumpston M, Li T, Page MJ, Welch VA (editors), Cochrane

[15] Sterne JA, Hernan MA, Reeves BC, et al. ROBINS-I: a tool for assessing risk of bias in non-randomised studies of interventions. BMJ. 2016;355:i4919. 27733354 10.1136/bmj.i4919 PMC5062054

[16] Guyatt G, Oxman AD, Akl EA, et al. GRADE guidelines: 1 Introduction-Grade evidence profiles and summary of findings tables. J Clin Epidemiol. 2011;64(4):383–94. 21195583 10.1016/j.jclinepi.2010.04.026

[17] Al-Dorzi HM, Alruwaita AA, Marae BO, et al. Incidence, risk factors and outcomes of seizures occurring after craniotomy for primary brain tumor resection. Neurosci Riyadh. 2017;22(2):107. 10.17712/nsj.2017.2.20160570 PMC5726815 28416781

[18] Yeap M-C, Chen C-C, Liu Z-H, et al. Postcranioplasty seizures following decompressive craniectomy and seizure prophylaxis: a retrospective analysis at a single institution. J Neurosurg. 2018;131(3):936–40. 30239312 10.3171/2018.4.JNS172519

[19] Lavergne P, Labidi M, Brunet M-C, et al. Efficacy of antiseizure prophylaxis in chronic subdural hematoma: a cohort study on routinely collected health data. Journal Neurosurg. 2019;132:28–48. 10.3171/2018.9.JNS182092 30660118

[20] Garbossa D, Panciani PP, Angeleri R, et al. A retrospective two-center study of antiepileptic prophylaxis in patients with surgically treated high-grade gliomas. Neurol India. 2013;61(2):131–7. 23644311 10.4103/0028-3886.111118

[21] Battaglia F, Plas B, Melot A, et al. Is there any benefit from short-term perioperative antiepileptic prophylaxis in patients with chronic subdural haematoma? A retrospective controlled study. Neurochirurgie. 2015;61(5):324–8. 26256569 10.1016/j.neuchi.2015.06.004

[22] Lee ST, Lui TN, Chang CN, et al. Prophylactic anticonvulsants for prevention of immediate and early postcraniotomy seizures. Surg Neurol. 1989;31(5):361. 2711309 10.1016/0090-3019(89)90067-0

[23] Wu AS, Trinh VT, Suki D, et al. A prospective randomized trial of perioperative seizure prophylaxis in patients with intraparenchymal brain tumors. J Neurosurg. 2013;118(4):873. 23394340 10.3171/2012.12.JNS111970 PMC4083773

[24] North JB, Penhall RKH. A. phenytoin and postoperative epilepsy. A double-blind study. J Neurosurg. 1983;58(5):672–7. 6339686 10.3171/jns.1983.58.5.0672

[25] Liang S, Ding P, Zhang S, Zhang J, Wu Y. Prophylactic levetiracetam for seizure control after cranioplasty: a multicenter prospective controlled study. World Neurosurg. 2017;102:284. 28315449 10.1016/j.wneu.2017.03.020

[26] Foy PM, Chadwick DW, Rajgopalan N, Johnson AL, Shaw MD. Do prophylactic anticonvulsant drugs alter the pattern of seizures after craniotomy? J Neurol Neurosurg Psychiatry. 1992;55(9):753. 1402964 10.1136/jnnp.55.9.753 PMC1015096

[27] Pradhanang A, Sedain GSSKSMR. Prophylactic use of antiepileptic drug (phenytoin) in preventing early postoperative seizure in patients with chronic subdural hematoma: a randomized control trial. Indian J Neurosurg. 2019;8(3):168.

[28] Kale A. Prophylactic anticonvulsants in patients undergoing craniotomy: a single-center experience. Med Sci Monit. 2018;24:2578–82. 29700277 10.12659/MSM.908717 PMC5941984

[29] Wu AS, Trinh VT, Suki D, et al. A prospective randomized trial of perioperative seizure prophylaxis in patients with intraparenchymal brain tumors: Clinical article. J Neurosurg. 2013;118(4):873. 23394340 10.3171/2012.12.JNS111970 PMC4083773

[30] Battaglia F, Plas B, Melot A, et al. Is there any benefit from short-term perioperative antiepileptic prophylaxis in patients with chronic subdural haematoma? A Retrospect Control Study Neurochirurgie. 2015;61(5):324–8. 10.1016/j.neuchi.2015.06.004 26256569

[31] Freund B, Probasco JC, Ritzl EK. Seizure incidence in the acute postneurosurgical period diagnosed using continuous electroencephalography. J Neurosurg. 2018;27:1–7. 10.3171/2018.1.JNS171466 30067470

[32] Tabaeizadeh M, Aboul Nour H, Shoukat M, et al. Burden of epileptiform activity predicts discharge neurologic outcomes in severe acute ischemic stroke. Neurocrit Care. 2020;32(3):697–706. 32246435 10.1007/s12028-020-00944-0 PMC7416505

[33] Gaynor JW, Jarvik GP, Gerdes M, et al. Postoperative electroencephalographic seizures are associated with deficits in executive function and social behaviors at 4 years of age following cardiac surgery in infancy. J Thorac Cardiovasc Surg. 2013;146(1):132–7. 23768805 10.1016/j.jtcvs.2013.04.002 PMC4617776

[34] Rabinstein AA, Chung SY, Rudzinski LA, Lanzino G. Seizures after evacuation of subdural hematomas: incidence, risk factors, and functional impact. J Neurosurg. 2010;112(2):455–60. 19698050 10.3171/2009.7.JNS09392

[35] Fang T, Valdes E, Frontera JA. Levetiracetam for seizure prophylaxis in neurocritical care: a systematic review and meta-analysis. Neurocrit Care. 2022;36(1):248–58. 34286461 10.1007/s12028-021-01296-z

[36] North JB, Penhall RK, Hanieh A, Frewin DB, Taylor WB. Phenytoin and postoperative epilepsy. A double-blind study. J Neurosurg. 1983;58(5):672–7. 6339686 10.3171/jns.1983.58.5.0672

[37] Fuller KL, Wang YY, Cook MJ, Murphy MA, D’Souza WJ. Tolerability, safety, and side effects of levetiracetam versus phenytoin in intravenous and total prophylactic regimen among craniotomy patients: a prospective randomized study. Epilepsia. 2013;54(1):45–57. 22738092 10.1111/j.1528-1167.2012.03563.x

[38] Iuchi T, Kuwabara K, Matsumoto M, et al. Levetiracetam versus phenytoin for seizure prophylaxis during and early after craniotomy for brain tumours: a phase II prospective, randomised study. J Neurol Neurosurg Psychiatry. 2015;86(10):1158. 25511789 10.1136/jnnp-2014-308584

[39] Milligan TA, Hurwitz S, Bromfield EB. Efficacy and tolerability of levetiracetam versus phenytoin after supratentorial neurosurgery. Neurology. 2008;71(9):665. 18725591 10.1212/01.wnl.0000324624.52935.46

[40] Hohne J, Schebesch K-M, Ott C, Brawanski A, Lange M. The risk of hypotension and seizures in patients receiving prophylactic anti-epileptic drugs for supratentorial craniotomy. J Neurosurg Sci. 2018;62(4):418. 27854111 10.23736/S0390-5616.16.03826-1

[41] Shaw MD, Foy P, Chadwick D. The effectiveness of prophylactic anticonvulsants following neurosurgery. Acta Neurochir. 1983;69(3–4):253–8. 6681517 10.1007/BF01401812

[42] Lee YJ, Kim T, Bae SH, et al. Levetiracetam compared with valproic acid for the prevention of postoperative seizures after supratentorial tumor surgery: a retrospective chart review. CNS Drugs. 2013;27(9):753. 23921717 10.1007/s40263-013-0094-6

[43] Rahman M, Eisenschenk S, Melnick K, et al. Duration of prophylactic levetiracetam after surgery for brain tumor: a prospective randomized trial. Neurosurgery. 2023;92(1):68–74. 36156532 10.1227/neu.0000000000002164

[44] Abdolkarim Rahmanian AMS, Derakhshan N, Ziarati NK, Owji SH, Shahraki HR. Long- versus short-term seizure prophylaxis after craniotomy for clipping in aneurysmal subarachnoid hemorrhage; a retrospective cohort study. Arch Neurosci. 2019;6(2):68108.

[45] Strein M, Holton-Burke JP, Stilianoudakis S, et al. Levetiracetam-associated behavioral adverse events in neurocritical care patients. Pharmacotherapy. 2023;43(2):122–8. 36606737 10.1002/phar.2760

