# Supplementary material for: Guidelines for Seizure Prophylaxis in Patients Undergoing Supratentorial Neurosurgery: A Statement for Healthcare Professionals from the Neurocritical Care Society
Source: Neurocrit Care. 2026 May 5;45(1):34–57. doi: 10.1007/s12028-026-02522-2 (PMC13369652; doi:10.1007/s12028-026-02522-2)
Supplement: Supplementary file 1 — Supplementary file1 (PDF 1774 KB) [file 12028_2026_2522_MOESM1_ESM.pdf]

PRISMA 2009 Flow Diagram

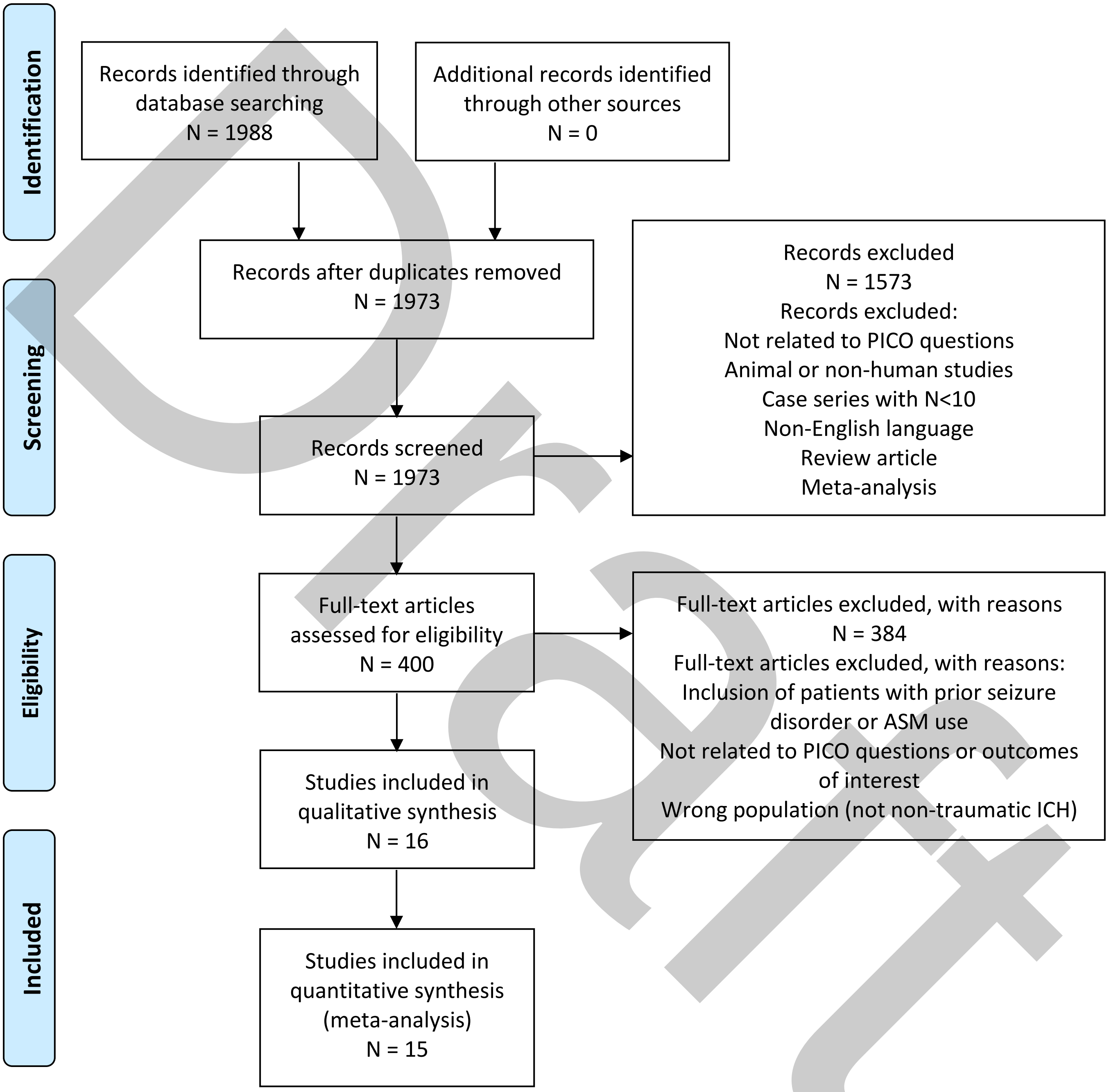

Draft

From: Moher D, Liberati A, Tetzlaff J, Altman DG, The PRISMA Group (2009). *P*referred *R*eporting *I*tems for *S*ystematic Reviews and *M*eta-*A*nalyses: The PRISMA Statement. PLoS Med 6(6): e1000097. doi:10.1371/journal.pmed1000097

For more information, visit www.prisma-statement.org.

Supplemental Table 1: Medical Librarian Search Strategy. To 'read' the search strategy - there is a MeSH term (has a '/'), if available followed by synonyms for the MeSH term (has .tw,kf). If there is no MeSH term, then the synonyms come from the relevant EMTREE term in Embase. Search conducted using Ovid MEDLINE(R) ALL 1946 to July 07, 2020

#	Searches	Results
1	Anticonvulsants/	51067
2	("anti convulsant agent?" or "anti epileptic agent?" or "anti epileptic drug?" or "anticonvulsant?" or "anticonvulsive agent?" or "anticonvulsive drug?" or "anticonvulsivum" or "antiepileptic agent?" or "antiepileptic barbiturate?" or "antiepileptic drug?" or antiepileptics or "antiepileptiform drug?").tw,kf.	44053
3	(brivaracetam or briviact or brivlera or nubriveo or rikelta or "ucb 34714" or "ucb34714").tw,kf.	268
4	Cannabidiol/	1610
5	(cannabidiol or epidiolex or epidyolex or "gwp 42003p" or "gwp42003p" or nabidiolex).tw,kf.	2861
6	Carbamazepine/	11105
7	(amizepin or amizepine or atretol or biston or calepsin or camapine or carbadac or carbamazepin or carbamazepine or carbategral or carbatol or carbatrol or carbazene or carbazep or carbazepin or carbazina or carmaz or carnexiv or carpaz or carzepin or carzepine or clostedal or epileptol or epimax or epitol or equetro or espa-lepsin or finlepsin or foxalepsin or "g 32883" or "g32883" or hermolepsin or karbamazepin or kodapan or lexin or mazepine or mazetol or neugeron or neurotol or neurotop or nordotol or panitol or servimazepin or sirtal or "spd 417" or "spd417" or tardotol or taver or tegol or tegral or tegretal or Tegretol or tegrital or telesmin or temporol or teril or timonil).tw,kf.	15445
8	(cenobamate or carbamate or "ykp 3089" or "ykp3089").tw,kf.	7844
9	Divalproex/	12533
10	("Abbott 50711" or depakote or divalproex or epival or semisodium valproate or valproate semisodium or valprolek).tw,kf.	924
11	Ethosuximide/	948
12	(asamid or "ci 366" or "ci366" or "cn 10,395" or "cn 10395" or "cn10,395" or "cn10395" or emeside or ethosuccimid or ethosuccimide or ethosuccinimide or ethosuximide or ethylmethylsuccimide or ethylsuximide or ethymal or ethymal or etosuximida or "h 490" or "h490" or mesentol or "nsc 64013" or "nsc64013" or	1655

Draft

	pemal or petimid or petinimid or petinimide or petnidan or "pm 671" or "pm671" or pyknolepsin or pyknolepsinum or ronton or simatin or succinutin or sucsilep or suksilep or suxilep or suximal or suxinutin or zarondan or zarontin).tw,kf.	
13	Felbamate/	400
14	(adipomin or "ahr 965" or "ahr965" or dimafen or fenfluramide or fenfluramin or fenfluramine or fenured or isomeride or kataline or minifage or moderex or obedrex or pesos or phenfluoramine or ponderal or ponderax or ponderex or pondimin or ponflural or rotondin or "val 1177" or "val1177").tw,kf.	4724
15	(fosphenytoin or "acc 9653" or "acc9653" or cerebyx or "ci 982" or "ci982" or fosphenytoin or phosphenytoin sodium or prodilantin).tw,kf.	492
16	Gabapentin/	3761
17	(apogabapentin or "ci 945" or "ci945" or convalis or dineurin or gabalept or "gabaliquid geriasan" or gabapentin or gabatin or gantin or "go 3450" or "go3450" or "goe 3450" or "goe3450" or gralise or kaptin or keneil or neurontin or neurotonin or nupentin).tw,kf.	6389
18	Lacosamide/	498
19	("add 234037" or "add234037" or erlosamide or harkoseride or lacosamide or "spm 927" or "spm927" or vimpat).tw,kf.	878
20	Lamotrigine/	3067
21	("bw 430c" or "bw 430c78" or "bw430c" or "bw430c78" or crisomet or labileno or labileno or lambipol or lamepil or lamictal or lamictin or lamiktal or lamitrin or lamodex or lamogine or lamotrigin or lamotrigine or lamotrix or medotrigin or neurium or "seizal (drug)").tw,kf.	5307
22	Levetiracetam/	2136
23	(elepsia or etiracetam or keppra or kopodex or levetiracetam or "lo 59" or "lo59" or matever or spritam or "ucb 6474" or "ucb l 059" or "ucb l059" or "ucb l060" or "ucb6474").tw,kf.	3855
24	Oxcarbazepine/	983
25	("gp 47680" or "gp47680" or "kin 493" or "npc 04" or "npc04" or oxcarbazepine or oxocarbazepine or oxrate or oxtellar or timox or "tri 476" or "tri476" or trileptal or trileptin).tw,kf.	1972
26	Pentobarbital/	10156

Draft

27	(auropan or barpental or diabutal or dorsital or embutal or etaminal or ethaminal or euthanyl or euthesate or isoamytal or iturate or mebubarbital or mebumal or mebumalum or medinox mono or mintal or napental or narcovet or nembutal or palapent or pentabarbitone sodium or pental or pentobarbilake or pentobarbital or pentobarbitalum or pentobarbitone or pentone or pentyl or praecicalm or sagatal or sedalixir or sombutol or somnopentyl or somnotol or sopental or sotyl or vetbutal).tw,kf.	19353
28	Phenobarbital/	17953
29	(adonal or aephenal or agrypnal or alepsal or amylofene or andral or aparoxal or aphenylbarbit or aphenyletten or atrofen or austrominal or barbapil or barbellen or barbenyl or barbilettae or barbilixir or barbinal or barbiphen or barbiphenyl or barbivis or barbonal or barbonalett or barbophen or bardorm or bartol or bialminal or calmetten or calminal or carbronal or cardenal or cemalonal or codibarbital or coronaletta or cratecil or damoral or dezibarbitur or dormina or dormiral or dromural or ensobarb or ensodorm or epanal or epidorm or epilol or episedal or epsylone or eskabarb or etilfen or euneryl or fenbital or fenemal or fenobarbital or fenolbarbital or fenosed or fenylettae or gardenal or gardenale or gardepanyl or glysoletten or haplopan or haplos or helional or hennoletten or hypnaletten or hypno tablinetten or hypnogen fragner or hypnolone or hypnotal or hypnotalon or hysteps or lefebar or leonal or lephebar or lepinal or lethyl or linasen or liquital or lixophen or lubergal or lubrokal or lumesettes or lumesyn or luminal or luminale or luminaletas or luminalette or luminaletten or luminalettes or luminalum or lumofridetten or luphenil or luramin or menobarb or molinal or neurobarb or nirvonal or noptil or nova pheno or nunol or parkotal or pharmetten or phen bar or phenaemal or phenemal or phenethylbarbital sodium or phenobal or phenobarb or phenobarbital or phenobarbiton or phenobarbitone or phenobarbitural or phenobarbyl or phenonyl or phenotal or phenoturic or phenoyl or phenyl ethyl barbituric acid or phenylbarbital or phenylethyl barbituric acid or phenylethylbarbituric acid or phenylethylmalonyl urea or phenylethylmalonylurea or phenyletten or phenyral or polcominal or promptonal or seda tablinen or sedabar or sedicat or sedizorin or sedlyn or sedofen or sedonal or sedonettes or seneval or sevenal or sombutol mcclung or somnolens or somnoletten or somnosan or somonal or spasepilin or starifen or starilettae or stental or teolaxin or theolaxin or triabarb	65617

Draft

	or tridezibarbitur or "uni-feno" or versomnal or wakobital or zadoletten or zadonal).tw,kf.	
30	Phenytoin/	13464
31	(alepsin or aleviatin or antilepsin or antisacer or cansoin or citrullamon or comital or cumatil or danten or dantoin or denyl or "di hydan" or difenin or difetoin or differenin or difhydan or dihydan or dilantin or dintoin or dintoina or diphantoin or diphantoine or diphedal or diphedan or "di-phen" or diphenin or diphenine or diphentoin or diphenyl hydantoin or diphenylan or diphenyldantoin or diphenylhydantoin or diphenytoin or ditoin or ditomed or dlphenylhydantoin sodium or ekko or epamin or epanutin or epelin or "epilan d" or epilantin or epileptin or eptal or eptoin or felantin or fenantoin or fenitoin or fenytoin or fenytoine or hidanil or hidantal or hydantin or hydantinal or hydantoinal or hydantol or idantoin or lehydan or lepitoin or minetoin or neosidantoina or phenhydan or phenhydane or phenilep or phentytoin or phenybin or phenydan or phenydantin or phenytek or phenytex or phenytoin or phenytoine or phenytoinum or phenytonium or pyoredol or sanepil or sodantoin or sodanton or sodium diphenylhydantoinate or solantoin or solantyl or tacosal or vasilcon or zentropil).tw,kf.	14977
32	Pregabalin/	1988
33	("148553-50-8" or "ci 1008" or "ci1008" or lyrica or "pd 144723" or "pd144723" or pregabalin).tw,kf.	3855
34	Primidone/	1297
35	(cyral or desoxyphenobarbital or desoxyphenobarbitone or hexadiona or lepsiral or liskantin or majsolin or midone or misodine or mizodin or mutigan or mylepsin or mylepsinum or mysolin or mysoline or neurosyn or primaclone or primadone or primidon holsten or primidone or prysoline or resimatil or sertan).tw,kf.	1269
36	Thiopental/	6840
37	(anesthal or bomathal or farmotal or hypnostan or intraval or leopental or nesdonal or penthiobarbital or penthotal or pentothal or pharmothal or ravonal or "rp 245" or "rp245" or sodipental or sodium pentothal or sodium thiopental or thiomebumal or thionembutal or thionyl or thiopental or thiopentalbarbital or thiopentemal or thiopenthal or thiopentobarbital or thiopentone or thiotal or thiothal or "tio pentemal" or "tiobarbital braun" or "tiopental sodico" or trapanal).tw,kf.	8541

Draft

38	Tiagabine/	521
39	("nnc 05 0328" or "nnc 328" or "no 05 0328" or "no 05 0329" or "no 328" or "no 329" or gabitril or tiabex or tiagabine).tw,kf.	939
40	Topiramate/	2738
41	(acomicil or ecuram or epiramat or epitomax or epitoram or erravia or etopro or fagodol or jadix or lusitrax or maritop or "mcn 4853" or "mcn4853" or "mcn-4853" or oritop or piraleps or pirantal or pirepil or qudexy or "ramas (drug)" or "rwj 17021" or "rwj 17021-000" or "rwj17021" or "rwj17021-000" or sincronil or talopam or tiramat or topaben or topamac or topamax or topepsil or topibrain or topilek or topimark or topimax or topiramat or topiramate or topiramato or topiratore or topit or toramat or torlepta or trokendi or "usl255").tw,kf.	4666
42	Trimethadione/	490
43	(absentol or edion or epidione or mino aleviatin or petidion or ptimal or tridione or trimethadion or trimethadione or trimetin or trioksal or troxidone).tw,kf.	465
44	Valproic Acid/	12533
45	("abbott 44090" or absenor or "alpha propylvalerate" or "alpha propylvaleric acid" or apilepsin or atemperator or "convival chrono" or convulex or convulsofin or delepsine or depacon or depakene or depakin or depakine or depakote or depalept or deprakine or diplexil or "dipropyl acetate" or "dipropyl acetic acid" or dipropylacetate or "dipropylacetatic acid" or "dipropylacetic acid" or diprosin or divalproex or epilam or epilex or epilim or episenta or "epival cr" or ergenyl or espa valept or everiden or goilim or hexaquin or "kw 6066 n" or labazene or leptilan or leptilanil or micropakine or mylproin or "myproic acid" or orfil or orfiril or orlept or petilin or "propylisopropylacetic acid" or propymal or stavzor or "valberg pr" or valcote or valepil or valeptol or valerin or "valhel pr" or valoin or valpakine or valparin or valporal or valprax or valpro or valproat or valproate or valprodura or "valproic acid" or valprosid or valprotek or valsup or vupral).tw,kf.	18173
46	Vigabatrin/	1587
47	(kigabeq or "mdl 71,754" or "mdl 71754" or "rmi 71754" or "rmi 71890" or sabril or sabrilex or vigabatrin or vigadrone).tw,kf.	1927
48	Zonisamide/	704
49	("68291-97-4" or "68291-98-5" or "Ad 810" or "ad810" or "ci 912" or "ci912" or excegran or excemid or zonegran or zonisamide).tw,kf.	1464
50	or/1-49 [Antiseizure Antiepileptic Drugs]	220784

Draft

51	Seizures/pc	3856
52	exp epilepsy/pc	1546
53	Pre-Exposure Prophylaxis/	2274
54	(prophylactic* or prophylaxi or prophylaxis).tw,kf.	164946
55	(preventive medication? or preventive therap* or preventive treatment? or protective therap*).tw,kf.	10949
56	(protect* adj3 brain).tw,kf.	7076
57	neuroprotect*.tw,kf.	64492
58	("no history" adj5 (epileps* or seizure?)).tw,kf.	271
59	or/51-58 [Seizure/Epilepsy prevention]	248998
60	50 and 59 [Antiseizure Antiepileptic Drugs AND Seizure/Epilepsy prevention]	8434
61	animals/ not humans.sh.	4682746
62	60 not 61	5202
63	craniotomy/ or decompressive craniectomy/ or trephining/	15517
64	(craniectomies or craniectomy or craniotom* or postcraniectom* or postcraniotom*).tw,kf.	18525
65	(trepanation? or trepanning? or trephination? or trephining?).tw,kf.	1992
66	or/63-65	28000
67	exp Supratentorial Neoplasms/	29101
68	supratentorial.tw,kf.	7852
69	or/67-68	35685
70	66 and 69 [supratentorial craniotomy]	1707
71	62 and 70 [Antiseizure Antiepileptic AND supratentorial craniotomy]	31
72	limit 71 to "all child (0 to 18 years)"	9
73	(adolescent or children).tw. or child, preschool.sh.	1617577
74	71 and 73	4
75	71 not (72 or 73)	22
76	limit 71 to "all adult (19 plus years)"	17
77	adult.mp. or middle aged.sh. or age*.tw.	9106473
78	71 and 77	21
79	75 or 76 or 78	30
80	limit 79 to english language	29

Draft

RCT

Study	Type of Study	Comparison	Outcome	Result	Risk of Bias in the randomization process	Risk of bias for intended interventions	Risk of Bias related to outcome data	Risk of Bias in Measurement of Outcome	Risk of bias related to the reported result	Overall Risk of Bias
Foy 1992	Individually-randomized parallel-group trial	Phenytoin vs. No phenytoin	New Seizure	PHT 42/105 vs. No PHT 25/59	Low	Low	Low	Some concern	Low	Some concern
Lee 1989	Individually-randomized parallel-group trial	Phenytoin vs. No phenytoin	New Seizure	PHT 2/189 vs. No PHT 13/185	Low	Low	Low	Some concern	Low	Some concern
Liang 2017	Individually-randomized parallel-group trial	Levetiracetam vs. No ASD	New Seizure	2/97 LEV with seizure at 24 weeks; 17/100 control with seizure at 24 weeks	Low	Low	Low	Some concern	Low	Some concern
Liang 2017	Individually-randomized parallel-group trial	Levetiracetam vs. No ASD	MRS GOSE other ADL	ADL score improvement 4.55 in LEV, 3.98 in control (no difference)	Low	Low	Low	Low	Low	Low
Liang 2017	Individually-randomized parallel-group trial	levetiracetam vs. No ASD	Adverse Events	ASD 22/95 vs. No ASD 19/83	Low	Low	Low	Low	Low	Low
Liang 2017	Individually-randomized parallel-group trial	Levetiracetam vs. No ASD	Cognitive Function	Memory quotient improved 3.72 in LEV, 3.19 in controls (no difference)	Low	Low	Low	Low	Low	Low
Luchi 2015	Individually-randomized parallel-group trial	levetiracetam vs. pheyntoin	New Seizure	Early: LEV 1/73 vs. PHT 11/73	Some concern	Some concern	Some concern	Some concern	Low	Some concern
Fuller 2013	Cluster-randomized parallel-group trial	levetiracetam vs. phenytoin	New Seizure	Early: LEV 0/36 vs. PHT 6/38 PHT	Low	Low	Low	Some concern	Low	Some concern
Fuller 2013	Cluster-randomized parallel-group trial	levetiracetam vs. phenytoin	Adverse Events	At 3 days: 1/36 LEV with side effect, 4/38 PHT with side effect	Low	Low	Low	Low	Low	Low
North 1983	Individually-randomized parallel-group trial	Phenytoin vs. No phenytoin	New Seizure	Late: ASD 18/140 PHT vs. No ASD 26/141	Low	Low	Low	Low	Low	Low
North 1983	Individually-randomized parallel-group trial	Phenytoin vs. No phenytoin	Adverse Events	ASD 12/140 vs. 3/141	Low	Low	Low	Low	Low	Low
Wu 2013	Individually-randomized parallel-group trial	Phenytoin vs. No phenytoin	Adverse Events	ASD 11/62 vs. 0/61	Low	Low	Low	Some concern	Low	Some concern
Wu 2013	Individually-randomized parallel-group trial	Phenytoin vs. No phenytoin	New Seizure	Early: ASD 6/62 vs No ASD 5/61<br>Late: ASD 9/62 vs. No ASD6/61	Low	Low	Low	Some concern	Low	Some concern

ASD = Antiseizure Drug
LEV = Levetriacetam
LCS = Lacosamide
PB = phenobarbital
PHT = Pheytoin
VPA = Valproic Acid

Draft

NonRCT

Study	Type of Study	Comparison	Outcome	Result	Bias due to Confounding	Bias in Selection of participants into the study	Bias in classification of interventions	Bias due to deviations from intended interventions	Bias due to missing data	Bias in Measurement of outcomes	Bias in selection of the reported result	Overall Risk of Bias
Al-Dorzi 2017	Retrospective Cohort	Post op ASDs vs. No ASDs	New Seizure	ASD 1/32 vs. No ASD 11/92	Critical	Moderate	Low	Low	Low	Low	Moderate	Critical
Battaglia 2015	Retrospective Cohort	ASDs vs. No ASDs	New Seizure	ASD 2/48 vs. No ASD 3/51	Moderate	Low	Low	Low	Low	Low	Low	Moderate
Hohne 2018	Prospective Cohort	LEV vs. PHT	Adverse Events	LEV 0/40 vs. PHT 4/41	Serious	Low	Low	Low	Low	Low	Low	Serious
Kale 2018	Retrospective Cohort	ASD vs. None	New Seizure	Did not compare	Critical	Low	Low	Low	Low	Low	Low	Critical
Lavergne 2019	Retrospective Cohort	ASD vs. No ASD	New Seizure	ASD 8/30 vs. No ASD 5/90	Moderate	Low	Low	Low	Low	Low	Low	Moderate
Lee 2013	Retrospective Cohort	LEV vs. VPA	New Seizure	Early: LEV 4/51 vs. VPA 10/231<br>Late: LEV 0/51 vs. VPA 5/231	Critical	Low	Low	Low	Low	Low	Low	Critical
Lee 2013	Retrospective Cohort	LEV vs. VPA	Adverse Events	LEV 5/51 vs. 62/231	Critical	Low	Low	Low	Low	Low	Low	Critical
Milligan 2008	Retrospective Cohort	LEV vs. PHT	New Seizure	Early: LEV 1/105 vs. PHT 9/210	Serious	Serious	Low	Low	Moderate	Low	Low	Serious
Rahmanian 2019	Retrospective Cohort	PHT 1 month post op vs. PHT 3 months	New Seizure	Seizure: short 9/238 vs. long 6/160	Moderate	Moderate	Moderate	Low	Low	Low	Low	Moderate
Shaw 1983	Retrospective Cohort	Some people got ASD vs. No clear comparator	New Seizure	PHT 5/50 vs. CBZ 8/52	Critical	Serious	Serious	Critical	Low	Serious	Moderate	Critical
Yeap 2018	Retrospective Cohort	ASD (PHT, VPA, LEV) vs. no ASD use	New Seizure	Early: ASD 0/56 vs. No ASD 25/280<br>Late: ASD 16/56 vs. No ASD 48/280	Moderate	Moderate	Moderate	Moderate	Low	Low	Low	Moderate
Garbossa 2013	Retrospective Cohort	LEV vs. No ASD	New Seizure	Early: ASD 1/43 vs. 0/48<br>Late: ASD 7/43 vs. No ASD 9/48	Low	Low	Low	Moderate	Moderate	Low	Low	Moderate

ASD = Antiseizure Drug
CBZ = Carbamazepine
LEV = levitiracetam
PHT = phenytoin
VPA = valproic acid

Draft
